# Novel Chalcone Derivatives as Anti-*Leishmania infantum* Agents with Potential Synergistic Activity and In Silico Insights

**DOI:** 10.3390/antibiotics14111123

**Published:** 2025-11-07

**Authors:** Ana Letícia Monteiro Fernandes, Abraão Pinheiro Sousa, Delva Thyares Fonseca Lamec, Leonardo Lima Cardoso, Rosália Santos Ferreira, Shayenne Eduarda Ramos Vanderley, Petrônio Filgueiras Athayde-Filho, Gabriela Fehn Fiss, Tatjana Souza Lima Keesen

**Affiliations:** 1 Immunology Laboratory of Infectious Diseases (LABIDIC), Department of Cellular and Molecular Biology, Federal University of Paraiba, João Pessoa 58051-900, Brazil; amf.leticia@gmail.com (A.L.M.F.);; 2 Bioenergy and Organic Synthesis Research Laboratory (LPBS), Department of Chemistry, Federal University of Paraíba, João Pessoa 58051-900, Brazil

**Keywords:** acetamides, antileishmanial, flavonoids, synergy

## Abstract

**Background:** Visceral leishmaniasis (VL) is a neglected tropical disease with limited therapeutic options, often restricted by toxicity, high costs, and resistance. Chalcones are promising scaffolds for the development of antiparasitic agents. **Objectives:** This study aimed to synthesize novel acetamides derived from 4-hydroxychalcones and evaluate their antileishmanial activity, cytotoxicity, potential synergy with amphotericin B (AmB), and mechanisms of action through in silico analyses. **Methods:** Six chalcone–acetamides (**3a**–**c**, **4a**–**c**) were synthesized and characterized by IR, NMR, and HRMS. In vitro activity against *Leishmania infantum* promastigotes and axenic amastigotes was assessed by colorimetric assays. Cytotoxicity was tested in human erythrocytes and PBMCs. Synergy with AmB was analyzed by the combination index. Molecular docking targeted parasite enzymes, and ADMET tools predicted pharmacokinetic and safety profiles. **Results:** Phenyl-substituted derivatives (**3a**–**c**) were inactive, while cyclohexyl-substituted analogs (**4a**–**c**) were active. Compound **4b** displayed the strongest effect (IC_50_: 7.02 μM for promastigotes, 3.4 μM for amastigotes), with low cytotoxicity and high Selectivity Indices. In combination with AmB, compound **4b** reduced the effective dose (DRI: 2.87) and increased the therapeutic window. Docking revealed favorable interactions of compound **4b** with deubiquitinase DUB16 and tryparedoxin peroxidase I, suggesting enzyme inhibition. ADMET predictions supported good absorption and low toxicity. **Conclusions:** Compound **4b** demonstrated potent and selective antileishmanial activity, synergism with AmB, and predicted safety. These findings highlight chalcone derivative **4b** as a promising lead for future preclinical development in VL therapy.

## 1. Introduction

Visceral leishmaniasis (VL), caused by protozoan parasites of the *Leishmania* genus, remains one of the most neglected tropical diseases, with *Leishmania infantum* being the primary etiological agent in Brazil [1]. These parasites have a digenetic life cycle, alternating between the promastigote (flagellated) stage in the insect vector’s gut and the amastigote (internalized flagellum) form within phagolysosomes of vertebrate host phagocytic cells [2,3]. Transmitted by the bite of infected *Lutzomyia* sandflies, VL affected more than 70,000 people in the Americas between 2001 and 2023, with a mortality rate of 8% [4].

Current commercial drugs, including pentavalent antimonials, amphotericin B (AmB), and miltefosine, are plagued by severe drawbacks: high toxicity, escalating resistance, and prohibitive costs [5,6]. These limitations highlight the necessity for alternative compounds that combine efficacy, affordability, and improved safety profiles. Chalcones (1,3-diaryl-2-propen-1-ones) have emerged as promising candidates for antileishmanial drug development due to their structural simplicity, ease of synthesis, and broad-spectrum bioactivity. These α,β-unsaturated ketones serve as precursors to flavonoids and exhibit well-documented anti-inflammatory, antimicrobial, and antiparasitic effects [7,8,9]. Chemical modifications of the chalcone scaffold, such as the introduction of acetamide moieties—class of carboxamide acids—have been explored to optimize biological activity and pharmacological profiles [10]. Recent studies highlight the potential of synthetic chalcone derivatives to address the limitations of current therapies, particularly the issue of selective toxicity, which remains a significant challenge in VL treatment [11].

Moreover, synergistic approaches that combine natural or synthetic compounds with conventional drugs have been increasingly explored in various therapeutic contexts, including bacterial, fungal, and parasitic infections, as a strategy to enhance efficacy and reduce toxicity. In the case of leishmaniasis, this strategy has also garnered attention, with studies showing promising results [12,13,14]. Therefore, the study aimed to synthesize and characterize novel acetamides derived from 4-hydroxychalcones and evaluate their in vitro antileishmanial activity against *L. infantum* alongside a potential synergistic interaction with AmB.

## 2. Results

### 2.1. Synthesis of Chalcone–Acetamides

**Scheme 1:**
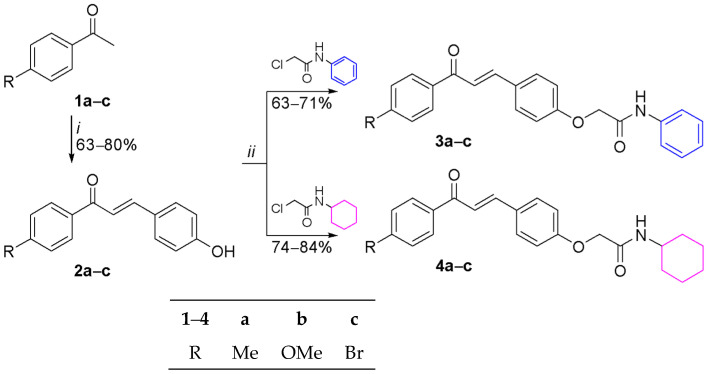
Synthetic route to obtain 4-hydroxychalcone derivatives (**2a**–**c**, **3a**–**c**, **4a**–**c**). Reagents and reaction conditions: (*i*) 4-hydroxybenzaldehyde, H_2_SO_4_, EtOH, 0–25 °C, 24 h; (*ii*) K_2_CO_3_, DMF, 80 °C, 6 h.

The synthetic route to obtain novel acetamides derived from 4-hydroxychalcones is outlined in Scheme 1. Chalcone–acetamides (**3a**–**c**, **4a**–**c**) were characterized by spectroscopic techniques of IR, ^1^H, and ^13^C NMR (Nuclear Magnetic Resonance), and HRMS (High-Resolution Mass Spectrometry) analysis.

4-Hydroxychalcones (**2a**–**c**) were prepared via a Claisen–Schmidt condensation reaction of 4-hydroxybenzaldehyde with acetophenones (**1a**–**c**) in the presence of concentrated sulfuric acid, yielding 63-80% of the product. Next, chalcone–acetamides (**3a**–**c**, **4a**–**c**) were obtained from an alkylation reaction of 4-hydroxychalcones with 2-chloro-*N*-phenylacetamide or 2-chloro-*N*-cyclohexylacetamide in the presence of potassium carbonate, in yields ranging from 63 to 84%.

In the NMR spectra of chalcone–acetamides, when compared to the signal of the methylene group Cl–CH_2_ from the acetamide precursors, the main signal confirming the formation of the products is that attributed to the methylene group O–CH_2_, due to the higher electronegativity of oxygen compared to chlorine, which leads to shifts to lower fields. Thus, in the ^1^H NMR spectra of chalcone–acetamides, the methylene hydrogens O–CH_2_ appeared as a singlet in the range of 4.50–4.67 ppm. Furthermore, the signal corresponding to OH from the 4-hydroxychalcone precursors was not observed. Finally, in the ^13^C NMR spectra of chalcone–acetamides, the methylene carbons O–CH_2_ appeared in the range of 67.5–67.7 ppm.

Regarding the IR spectra of chalcone–acetamides, when compared to their 4-hydroxychalcone precursors, the most significant changes were the absence of a broad band related to H–O, the emergence of H–N and C=O_amide_ bands at 3371–3393 and 1672–1687 cm^−1^, respectively, as well as an increase in symmetric/asymmetric stretching and in-plane/out-of-plane angular deformations related to C–O/N.

HRMS analyses confirmed the exact masses of the newly synthesized compounds (**3a**–**c**, **4a**–**c**) with errors of less than 5 ppm.

### 2.2. Assessment of Inhibitory Effects Against L. infantum

A screening was conducted to assess the anti-*L. infantum* activity of compounds **3a**–**c** and **4a**–**c** against promastigote forms. Viability analysis showed that compounds **3a**–**c** exhibited no inhibitory activity and were therefore excluded from further testing. In contrast, compounds **4a**–**c** inhibited *L. infantum* promastigotes in a dose-dependent manner with varying levels of activity.

**Figure 1:**
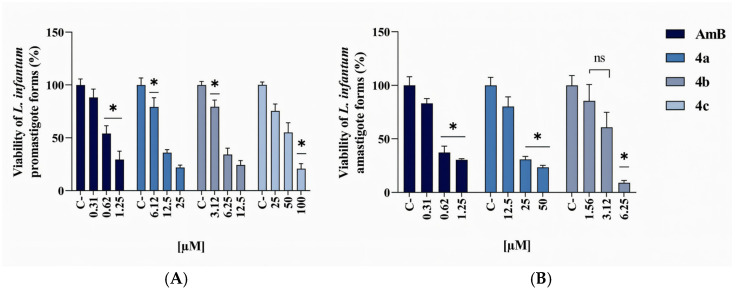
In vitro antileishmanial activity of AmB, **4a**, **4b,** and **4c** against *L. infantum* promastigotes (**A**) after 72 h treatment, and amastigotes (**B)** after 24 h treatment. The data represent the mean ± standard error of the mean (SEM) and were analyzed by one-way ANOVA, followed by the post hoc Tukey test. * *p* ≤ 0.05 and ns = non-significant.

Compound **4a** showed intermediate activity with a 50% inhibitory concentration (IC_50_) of 12.9 µM and a 95% Confidence Interval (CI) of 9.81–17.01 µM. At 25 µM, viability rates exhibited statistical differences when compared to 12.5 µM and 6.25 µM (Figure 1A). Viability data showed decreasing values from 100% at the lowest doses to 20.77% at the highest concentration, confirming its leishmanicidal effect. Compound **4b** proved more promising, with an IC_50_ of 7.02 µM (95% CI: 3.73–15.48 µM). The inhibition curve showed a marked response even at lower concentrations, with statistical significance between 3.12 µM and higher concentrations of 6.25 µM and 12.5 µM (Figure 1A). Mean viability values confirmed this tendency, with rates decreasing to 79.31% (3.12 µM), 34.17% (6.25 µM), and 24.31% (12.5 µM). These data indicate that compound **4b** has good efficacy even at moderate concentrations.

Compound **4c** exhibited limited activity, with significant inhibition only at higher concentrations (25–100 µM). The calculated IC_50_ was 45.8 µM (95% CI: 34.78–62.17 µM), indicating low leishmanicidal potency. The 25 µM concentration showed significant differences when compared to 50 µM and 100 µM (Figure 1A). The viability means corroborating these findings, with values of 100% at lower concentrations progressively decreasing to 21.93% at the highest concentration, indicating that only high doses of compound **4c** exert significant inhibitory effects on promastigotes. AmB displayed an IC_50_ of 0.87 µM (95% CI: 0.56–1.41 µM), confirming its activity against promastigotes.

In the amastigote analysis, which represents the clinically more relevant intracellular parasite stage, data revealed marked differences between compounds. Compound **4a** demonstrated leishmanicidal activity with an IC_50_ of 19.65 µM (95% CI: 11.9–33.2 µM). The 25 µM and 50 µM concentrations showed significant differences compared to 12.5 µM and the control (Figure 1B). Viability means decreased from 100% to 80.21% (12.5 µM), 30.77% (25 µM), and 23.51% (50 µM), demonstrating effects at higher doses.

Compound **4b** remained the top-performing compound, with an IC_50_ of 3.4 µM (95% CI: 2.99–3.9 µM), demonstrating significant potency. Statistically, 1.56 µM and 3.12 µM concentrations significantly differed from 6.25 µM (Figure 1B), while exhibiting no difference between them (ns). Mean viability values showed consistent decline from 100% to 85.46% (1.56 µM), 60.77% (3.12 µM), and 9.12% at 6.25 µM, confirming its high activity against amastigotes. Compound **4c** showed no detectable activity and was excluded after this analysis phase, reinforcing its low therapeutic potential. AmB maintained high potency against amastigotes, with an IC_50_ of 0.89 µM (95% CI: 0.43–2.11 µM).

### 2.3. Evaluation of Cytotoxicity in Human Cells and Selectivity Index Calculation

The cytotoxicity evaluation of compounds **4a**–**b** was conducted through two main parameters: red blood cell lysis and viability of Peripheral Blood Mononuclear Cells (PBMCs). The calculated 50% hemolytic concentration (HC_50_) for AmB was 193.4 μM (95% CI: 156.6–244.6 μM), demonstrating significant erythrocyte toxicity. In contrast, compounds **4a**–**b** did not reach HC_50_ thresholds within the tested concentrations.

**Figure 2:**
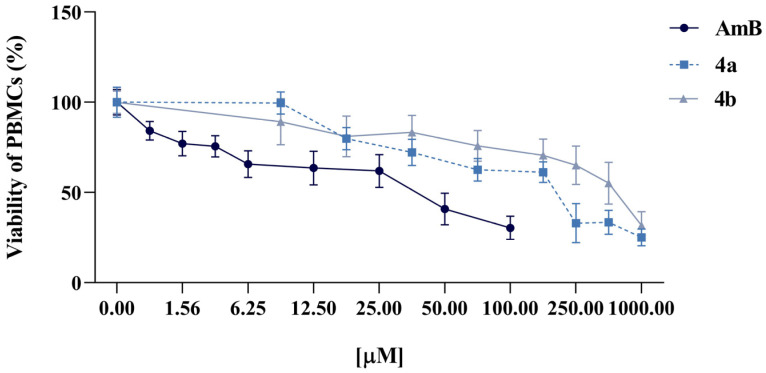
In vitro cytotoxicity of AmB, **4a,** and **4b** against human PBMCs after 24 h-treatment. The data represent the mean ± standard error of the mean (SEM).

Regarding PBMC viability, in the presence of AmB, viable cells progressively decreased: 61.89% at 25 μM, 40.82% at 50 μM, and 30.37% at 100 μM, demonstrating substantial cell damage even at moderate concentrations. Compound **4a** exhibited higher inhibition rates than compound **4b,** with viability percentages of 61.19% at 125 μM, decreasing to 32.97% at 250 μM and 33.43% at 500 μM. While demonstrating dose-dependent toxicity, the effects remained less severe than those of AmB, particularly at lower concentrations. For compound **4b**, no statistically significant differences were observed between the tested concentrations (25–100 μM) and the negative controls, indicating negligible cytotoxicity. Viability means were 65.06% at 250 μM, 55.16% at 500 μM, and 31.75% at 1000 μM. Though declining at the highest doses, values remained above critical viability thresholds, suggesting acceptable mononuclear cell toxicity (Figure 2). AmB had a 50% cytotoxic concentration (CC_50_) of 29.91 μM (95% CI: 19.1–53.4 μM), indicating a narrow therapeutic window. Compound **4b** demonstrated a higher CC_50_ of 505.4 μM (95% CI: 333.6–914.3 μM) and **4a** of 157.1 μM (95% CI: 105.6–241.8 μM).

The Selectivity Index (SI) was determined to evaluate the differential toxicity of the compounds toward mammalian host cells (PBMCs) versus the parasitic forms by calculating the ratio of the CC50 to the IC_50_. Both chalcones demonstrated selective activity; compound **4a** did not surpass the reference drug in selectivity, whereas compound **4b** exhibited a higher SI than AmB across both evaluated ratios (Table 1).

### 2.4. In Silico Insights

**Figure 3:**
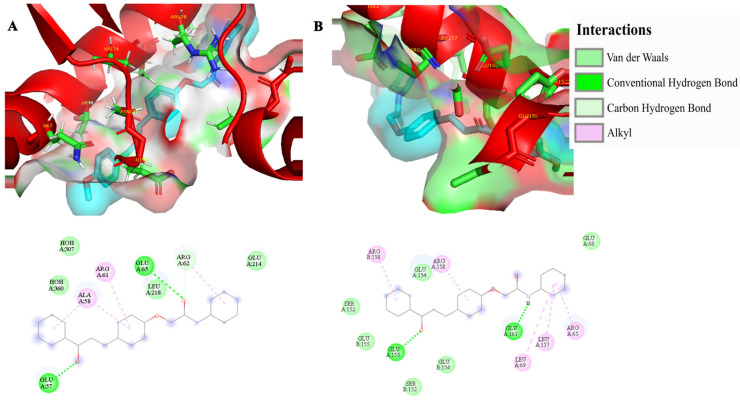
Three-dimensional representation of the molecular docking interactions between compound **4b** (ligand in cyan) and the target enzymes. (**A**) shows the highest affinity pose of compound **4b** in the binding site of the *Leishmania* Deubiquitinase, with the binding site surface and key residues highlighted. (**B**) displays the complex of compound **4b** with the *Leishmania* Tryparedoxin Peroxidase I. In both images, the protein structures are represented as red ribbons, and the amino acid residues as sticks. ARG = arginine; ALA = alanine; GLU = glutamic acid; LEU = leucine; SER = serine.

The binding affinity and potential molecular targets of the chalcone–acetamides (**4a**–**b**) were assessed through molecular docking against two key enzymes for parasite survival: the *Leishmania* Deubiquitinase and Tryparedoxin Peroxidase I. The analysis revealed favorable binding energies for the series, with compound **4b** demonstrating the most favorable within the series for the DUB16 (−5.73 kcal/mol), followed by **4a** (−4.92 kcal/mol). For the TXNPx, compound **4b** also showed the highest affinity (−5.005 kcal/mol), while compound **4a** exhibited a similar, albeit less favorable, binding energy (−4.78 kcal/mol) (Figure 3).

Detailed inspection (Figure 3) of the **4b**-DUB16 complex revealed that stabilizing conventional hydrogen bonds were observed with Glu57 and Glu65, while alkyl interactions occurred with Arg61 and Ala58. Additionally, Arg62 contributed through both carbon-hydrogen bond and alkyl contacts, highlighting a combination of polar and hydrophobic forces that anchor the ligand in the catalytic cleft. For the **4b**-TXNPx complex, conventional hydrogen bonds were formed with Glu155 and Glu161, while alkyl interactions involved Leu157, Leu69, and Arg65, in addition to two distinct alkyl contacts with Arg158. These residues collectively provide a favorable environment for the chalcone scaffold, combining hydrogen bonding and hydrophobic stabilization, which may interfere with the enzyme’s catalytic function.

The ProTox-II analysis classified both compounds **4a**–**b** as having low predicted toxicity (LD_50_ = 1600 mg·kg^−1^), which is in line with the absence of cytotoxicity in erythrocyte and PBMC assays. pkCSM predictions indicated that compounds **4a**–**b** may inhibit P-glycoprotein, while not affecting CYP2D6. Although this suggests potential metabolic liabilities, further validation is required. A predicted Caco-2 permeability value of 1.474 for compound **4a** and 0.651 for compound **4b** supports the possibility of adequate intestinal absorption.

SwissADME results indicated compliance with Lipinski’s Rule of Five, no violations being observed. Compound **4b** exhibited a Topological Polar Surface Area (TPSA, a descriptor related to drug transport) of 64.63 Å^2^, and a Consensus Log Po/w (a measure of lipophilicity) of 4.02. Compound **4a** had a TPSA of 55.4 Å^2^, and a Consensus Log Po/w of 4.35 (Table 2).

### 2.5. Synergistic Activity Between Compound ***4b*** and AmB

Compound **4b** was selected for further investigation of a potential synergistic activity with AmB due to its high Selectivity Index in the viability assay. At a fixed ratio of 1:5 (AmB:**4b**), the combination exhibited an IC_50_ of 2.54 μM. The combination index (CI) analysis revealed a dose-dependent interaction profile, with a slightly antagonistic effect at the ED50 (CI = 1.13), nearly additive at the ED75 (CI = 0.99), and synergistic at the ED_90_ (CI = 0.89).

**Figure 4:**
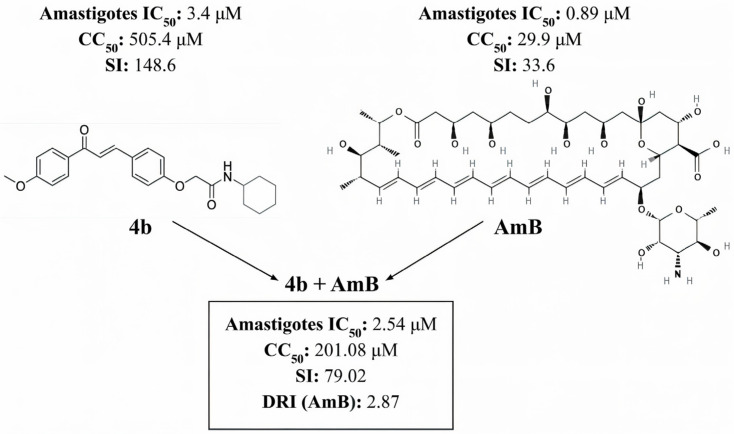
SI = Selectivity Index. CC_50_ = 50% Cytotoxic Concentration. IC_50_ = 50% Inhibitory Concentration. DRI = dose reduction index.

Although the CI at ED_50_ exceeded 1, the dose reduction index (DRI) values calculated at IC_50_ were 2.87 for AmB and 1.28 for compound **4b**. These values suggest the combination could achieve 50% antiparasitic efficacy at nearly one-third of AmB’s monotherapy dose, despite marginal antagonism at lower effects (ED_50_). Against PBMCs, the combination demonstrated a CC_50_ of 201.08 μM, indicating cytotoxicity only at concentrations above this level. The SI, calculated as the CC_50_/IC_50_ ratio for the combined compounds, of 79.02 was higher than that of AmB individually, suggesting that the combination provides a broader therapeutic window when used simultaneously with compound **4b** (Figure 4).

## 3. Discussion

The available treatments for VL face significant challenges, including high production costs, a history of toxicity, and the increasing emergence of drug resistance [5,6]. In light of these limitations, ongoing research efforts aim to identify safer and more effective therapeutic alternatives to conventional treatments. In this context, chalcone derivatives have attracted considerable interest due to their broad spectrum of biological activities [7,8].

Therefore, to explore new therapeutic candidates, a novel series of chalcone–acetamides (**3a**–**c** and **4a**–**c**) was synthesized via alkylation reactions as outlined in Scheme 1. The two series differ mainly in their *N*-substituent: phenyl in **3a**–**c** and cyclohexyl in **4a**–**c**, while sharing typical substituents at the para-position of the aromatic ring (Me, OMe, Br).

Following the successful synthesis and structural characterization of chalcone–acetamides, the compounds **3a**–**c** and **4a**–**c** were subjected to biological evaluation. Compounds **3a**–**c** exhibited no inhibitory activity against *L. infantum* promastigotes. In contrast, compounds **4a**–**c**, which incorporate a more hydrophobic and aliphatic cyclohexyl group, demonstrated a measurable leishmanicidal effect (Figure 1). This observation is consistent with previous reports highlighting the antimicrobial potential of cyclohexane-containing scaffolds [15], suggesting a potential structure–activity relationship in which the increased lipophilicity of the cyclohexyl moiety may enhance interaction with the parasite membrane or facilitate compound uptake. However, the lack of activity observed for the phenyl-substituted series (**3a**–**c**) remains inconclusive, suggesting that other physicochemical or steric factors may also influence their interaction with the parasite and warrant further investigation.

Compound **4c** exhibited only mild inhibition against promastigotes (IC_50_ = 45.8 µM; 34.78–62.17 µM) and no inhibitory effect against amastigote forms. Compounds **4a** and **4b** demonstrated potent activity against both promastigote (**4a**: 12.9 µM [9.81–17.01 µM]; **4b**: 7.02 µM [3.73–15.48 µM]) and amastigote forms (**4a**: 19.65 µM [11.9–33.2 µM]; **4b**: 3.4 µM [2.99–3.9 µM]). These results align with prior reports of the efficacy of chalcone derivatives against *L. infantum*, where IC_50_ values for amastigotes ranged from 2.24 to 63.89 µM [16] and 18.92–62.52 µM [17]. Similar activity has been documented against other *Leishmania* species, including *L. donovani* and *L. amazonensis* [18,19], underscoring the broad-spectrum potential of chalcone derivatives.

Additionally, Gomes et al. [20] evaluated the leishmanicidal activity of 32 chalcone-based compounds and their derivatives, reporting that halogen atoms were associated with reduced activity. This observation is consistent with the present findings, as compound **4c** contains a halogen element (bromine), whereas compounds **4a** and **4b** do not feature this modification.

In the cytotoxicity assays, compounds **4a** and **4b** (Figure 2) were initially assessed for their inhibitory activity against human erythrocytes and showed no hemolytic activity at any of the tested concentrations (100–0.78 µM). When tested against PBMCs, compounds **4a** and **4b** exhibited mild cytotoxicity, with CC_50_ values of 157.1 μM (95% CI: 105.6–241.8 μM) and 505.4 μM (95% CI: 333.6–914.3 μM), respectively. Notably, compound **4b** displayed a higher SI than AmB, the reference drug in this study, suggesting superior therapeutic safety (Table 1).

A study conducted by Santiago-Silva et al. [11] with a series of nine chalcones identified a compound with anti-*Leishmania amazonensis* activity against promastigote forms, internalized amastigote forms, and presented low in vitro cytotoxicity against murine macrophages with high SIs. Further supporting this, chalcone derivatives have shown low toxicity in both in vitro and in vivo infection models [21,22], reinforcing their potential.

Chalcone derivatives demonstrate biological effects through multiple mechanisms, including enzymatic inhibition and modulation of redox homeostasis in protozoan parasites. Previous studies have shown that chalcone derivatives can target cysteine proteases, trypanothione reductase, and other enzymes essential for parasite survival [8,9]. In recent years, in silico approaches, particularly molecular docking and ADMET prediction tools, have been increasingly applied to rationalize experimental findings and to identify plausible molecular targets for active compounds [23,24,25,26,27,28,29,30].

The in silico findings presented here support the biological activity of chalcone–acetamides by highlighting two critical enzymatic pathways as potential targets. Compound **4b**, which demonstrated the strongest in vitro leishmanicidal effect, also exhibited the most favorable docking energies against DUB16 and TXNPx (Figure 3). Deubiquitinases (DUBs) are increasingly recognized as central regulators of parasite viability, being essential for the growth of promastigotes and the differentiation of amastigotes [31,32]. The hydrogen bonds and hydrophobic contacts identified for compound **4b** within the catalytic pocket of DUB16 suggest that the compound could interfere with ubiquitination homeostasis, a process whose disruption has been shown to severely compromise *Leishmania* development and infectivity.

In parallel, TXNPx constitutes a key component of the parasite’s antioxidant defense system, neutralizing macrophage-derived hydrogen peroxide and preventing host–cell apoptosis [33]. In our docking simulations, compound **4b** formed stabilizing hydrogen bonds with Glu155 and Glu161, as well as multiple hydrophobic contacts, indicating a plausible mechanism for interfering with this redox defense pathway. This dual targeting of ubiquitination and redox homeostasis could explain the pronounced activity of compound **4b** against amastigotes, which rely heavily on both pathways to adapt to intracellular stress.

Moreover, the favorable ADMET predictions for compound **4b** (Table 2), including low cytotoxicity and compliance with Lipinski’s parameters, further reinforce its potential as a lead compound. Whilst docking results are exploratory and require biochemical validation, the convergence between experimental and computational findings strengthens the evidence that chalcone derivatives, particularly **4b**, can modulate critical parasite survival pathways.

Although compound **4b** exhibited promising results individually, the therapeutic potential of chalcone derivatives also supports the exploration of combination strategies aimed at enhancing treatment efficacy and reducing drug-associated toxicity. Therefore, the combined effects of compound **4b** and AmB were evaluated. The combination of compound **4b** and AmB at a 1:5 ratio demonstrated dose-dependent synergy, with a CI of 0.89 at ED_90_ and a 2.87-fold dose reduction index (DRI) for AmB, indicating enhanced efficacy at lower drug exposures. Chanmol et al. [14] evaluated the potential synergistic interaction between 8-hydroxyquinoline and AmB against *L. martiniquensis* promastigotes and intracellular amastigotes, identifying four combinations that exhibited synergistic effects in intracellular amastigotes without inducing toxicity in host cells. Similarly, it has been reported that the combination of AmB with triclabendazole (TCBZ), a benzimidazole used to treat fasciolosis, demonstrates synergy against both promastigotes and amastigotes of *L. amazonensis* [13].

Despite growing evidence of synergistic interactions between AmB and compounds from various chemical classes against *Leishmania* spp., combinations involving chalcone derivatives remain understudied in leishmaniasis. Analogous synergistic effects involving chalcones have only been primarily reported in other models, such as *Candida albicans* [34,35]. Altogether, these findings further highlight the relevance of exploring chalcone derivatives as potential antileishmanial agents and reinforce their promise in the development of alternative therapeutic strategies.

## 4. Materials and Methods

### 4.1. Reagents and Instrumentation

All common reagents were purchased from commercial suppliers (Sigma-Aldrich, Cajamar, SP, Brazil) and used without further purification. Compounds were synthesized at Laboratório de Pesquisa em Bioenergia e Síntese Orgânica (LPBS-UFPB), and melting points were measured using Microquímica equipment, model MQAPF-301 (Palhoça, Brazil). ^1^H and ^13^C Nuclear Magnetic Resonance (NMR) spectra were acquired from Laboratório Multiusuário de Caracterização e Análise (LMCA-UFPB), on Bruker Ascend (Coventry, UK) and Bruker (Billerica, MA, USA) spectrometers at 400 and 500 MHz for ^1^H, respectively, using 5 mm tubes at 298 K. Infrared (IR) spectra were acquired on a Shimadzu spectrometer, model IRPrestige-21 (Kyoto, Japan), at Laboratório de Síntese Orgânica Medicinal (LASOM-UFPB), using KBr pellets, and on a Shimadzu spectrometer (Kyoto, Japan), model MiRacleA (ZnSe), at Núcleo de Pesquisa e Extensão de Combustíveis e de Materiais (NPE-LACOM-UFPB), using the Attenuated Total Reflection (ATR) technique. High-Resolution Mass Spectrometry (HRMS) analyses were acquired at LMCA-UFPB, on a Shimadzu HPLC (Kyoto, Japan) coupled to a Bruker MicrOTOF II (Billerica, MA, USA), with an electrospray ion (ESI) source, and reported as *m*/*z* (relative intensity) for the molecular ion. Acquisition Parameters: Ion Polarity Positive, Capillary 4500 V, End Plate Offset −500 V, Nebulizer 4.0 Bar, Dry Heater 200 °C, Dry Gas 8.0 L·min^−1^, Divert Valve Waste.

### 4.2. General Procedure for Synthesis of 2-Chloro-N-Phenylacetamide and 2-Chloro-N-Cyclohexylacetamide

According to a method previously described by Costa et al. [36], with some adjustments, 2-chloro-*N*-phenylacetamide and 2-chloro-*N*-cyclohexylacetamide were obtained and recrystallized from an ethanol:water mixture (9:1) in yields of 86% and 70%, respectively. Full data for 2-chloro-*N*-phenylacetamide [36] and 2-chloro-*N*-cyclohexylacetamide [37] were found to be identical to those reported previously.

### 4.3. General Procedure for Synthesis of 4-Hydroxychalcones (***2a***–***c***)

According to a method previously described by Sousa et al. [38], 4-hydroxychalcones (**2a**–**c**) were obtained and recrystallized from an ethanol:water mixture (8:2) in yields ranging from 63% to 80%. Full data for 4-hydroxychalcones (**2a**–**c**) were found to be identical to those reported previously [38].

### 4.4. General Procedure for Synthesis of Chalcone–Acetamides (***3a***–***c***, ***4a***–***c***)

Based on a procedure reported by Tien et al. [39], with some adjustments, a mixture of 2-chloro-*N*-phenylacetamide or 2-chloro-*N*-cyclohexylacetamide (1.0 mmol), 4-hydroxychalcone (**2**, 1.0 mmol) and potassium carbonate (K_2_CO_3_, 1.2 mmol) in anhydrous dimethylformamide (DMF, 2 mL) was maintained under magnetic stirring at 80 °C for 6 h, which was monitored by thin-layer chromatography (TLC) in hexane:ethyl acetate (AcOEt) 1:1. Afterwards, the reactional mixture was cooled to room temperature, added to a beaker containing ice water (10 mL), and the precipitate was filtered with excess ice water. Compounds (**3a**–**c**, **4a**–**c**) were obtained and recrystallized from an ethanol:water mixture (9:1) in yields ranging from 63 to 84%. Full NMR, IR, and HRMS spectra for chalcone–acetamides (**3a**–**c**, **4a**–**c**) are available in Figures S1–S24 (Supplementary Material).

(*E*)-2-(4-(3-Oxo-3-p-tolylprop-1-enyl)phenoxy)-*N*-phenylacetamide (**3a**): Yield 71%; *Rf* 0.53 (hexane:AcOEt 1:1); pale yellow crystalline solid; m.p. 180–182 °C; ^1^H NMR (500 MHz, CDCl_3_) δ = 8.26 (s, 1H, NH), 7.93 (d, *J* = 8.2 Hz, 2H, H_Ar_), 7.76 (d, *J* = 15.6 Hz, 1H, H_β_), 7.64 (d, *J* = 8.9 Hz, 2H, H_Ar_), 7.59 (d, *J* = 7.5 Hz, 2H, H_Ar_), 7.45 (d, *J* = 15.6 Hz, 1H, H_α_), 7.36 (t, *J* = 8.0 Hz, 2H, H_Ar_), 7.30 (d, *J* = 7.9 Hz, 2H, H_Ar_), 7.16 (t, *J* = 7.4 Hz, 1H, H_Ar_), 7.03 (d, *J* = 8.9 Hz, 2H, H_Ar_), 4.64 (s, 2H, CH_2_), 2.43 (s, 3H, CH_3_) ppm; ^13^C NMR (126 MHz, CDCl_3_) δ = 189.8 (C=O_ketone_), 165.6 (C=O_amide_), 158.6, 143.6, 143.4, 136.7, 135.6, 130.3, 129.4, 129.3, 129.1, 128.6, 125.0, 120.8, 120.2, 115.2 (12 × C_Ar,_ C_β,_ C_α_), 67.5 (CH_2_), 21.6 (CH_3_) ppm; IR (ATR) ν = 3377 (H–N), 3058 (H–C=C), 2905, 2849 (H_alkanic_), 1684 (C=O_amide_), 1653 (C=O_ketone_), 1592, 1531, 1500 (C=C), 1243, 1218, 1163, 1071 (C–O/N), 973 (H*_trans_*), 820, 739, 691 (Ar) cm^−1^; HRMS (ESI) calculated for C_24_H_22_NO_3_ ([M + H]^+^) 372.1594, found 372.1595.

(*E*)-2-(4-(3-(4-Methoxyphenyl)-3-oxoprop-1-enyl)phenoxy)-*N*-phenylacetamide (**3b**): Yield 64%; *Rf* 0.51 (hexane:AcOEt 1:1); yellow crystalline solid; m.p. 156–158 °C; ^1^H NMR (400 MHz, CDCl_3_) δ = 8.21 (s, 1H, NH), 8.04 (d, *J* = 9.0 Hz, 2H, H_Ar_), 7.77 (d, *J* = 15.6 Hz, 1H, H_β_), 7.66 (d, *J* = 8.6 Hz, 2H, H_Ar_), 7.59 (d, *J* = 7.5 Hz, 2H, H_Ar_), 7.47 (d, *J* = 15.5 Hz, 1H, H_α_), 7.37 (t, *J* = 7.5 Hz, 2H, H_Ar_), 7.17 (t, *J* = 7.5 Hz, 1H, H_Ar_), 7.04 (d, *J* = 8.8 Hz, 2H, H_Ar_), 6.99 (d, *J* = 8.9 Hz, 2H, H_Ar_), 4.67 (s, 2H, CH_2_), 3.90 (s, 3H, CH_3_) ppm; ^13^C NMR (101 MHz, CDCl_3_) δ = 188.7 (C=O_ketone_), 165.8 (C=O_amide_), 163.5, 158.6, 143.1, 136.8, 131.3, 130.9, 130.4, 129.7, 129.3, 125.2, 120.8, 120.3, 115.3, 114.0 (12 × C_Ar,_ C_β,_ C_α_), 67.7 (CH_2_), 55.6 (CH_3_) ppm; IR (KBr) ν = 3371 (H–N), 3050, 3007 (H–C=C), 2920, 2834 (H_alkanic_), 1687 (C=O_amide_), 1650 (C=O_ketone_), 1600, 1532, 1502 (C=C), 1261, 1224, 1168, 1063, 1024 (C–O/N), 977 (H*_trans_*), 823, 756, 694 (Ar) cm^−1^; HRMS (ESI) calculated for C_24_H_22_NO_4_ ([M + H]^+^) 388.1543, found 388.1547.

(*E*)-2-(4-(3-(4-Bromophenyl)-3-oxoprop-1-enyl)phenoxy)-*N*-phenylacetamide (**3c**): Yield 63%; *Rf* 0.56 (hexane:AcOEt 1:1); pale yellow crystalline solid; m.p. 206–207 °C; ^1^H NMR (500 MHz, CDCl_3_) δ = 8.20 (s, 1H, NH), 7.88 (d, *J* = 8.7 Hz, 2H, H_Ar_), 7.79 (d, *J* = 15.7 Hz, 1H, H_β_), 7.67–7.64 (m, 4H, H_Ar_), 7.59 (d, *J* = 9.9 Hz, 2H, H_Ar_), 7.41–7.35 (m, 3H, H_α_, H_Ar_), 7.17 (t, *J* = 7.4 Hz, 1H, H_Ar_), 7.05 (d, *J* = 8.9 Hz, 2H, H_Ar_), 4.67 (s, 2H, CH_2_) ppm; ^13^C NMR (126 MHz, CDCl_3_) δ = 189.3 (C=O_ketone_), 165.7 (C=O_amide_), 158.9, 144.5, 137.1, 136.7, 132.0, 130.6, 130.1, 129.3, 129.3, 128.0, 125.2, 120.4, 120.3, 115.4 (12 × C_Ar,_ C_β,_ C_α_), 67.7 (CH_2_) ppm; IR (ATR) ν = 3389 (H–N), 3058 (H–C=C), 2910, 2838 (H_alkanic_), 1678 (C=O_amide_), 1653 (C=O_ketone_), 1593, 1543, 1506 (C=C), 1261, 1218, 1169, 1114, 1071, 1009 (C–O/N), 979 (H*_trans_*), 844, 820, 740, 691 (Ar) cm^−1^; HRMS (ESI) calculated for C_23_H_18_BrNaNO_3_ ([M + Na]^+^) 458.0362, found 458.0342.

(*E*)-*N*-Cyclohexyl-2-(4-(3-oxo-3-p-tolylprop-1-enyl)phenoxy)acetamide (**4a**): Yield 84%; *Rf* 0.52 (hexane:AcOEt 1:1); pale yellow solid; m.p. 152–153 °C; ^1^H NMR (500 MHz, CDCl_3_) δ = 7.93 (d, *J* = 8.2 Hz, 2H, H_Ar_), 7.76 (d, *J* = 15.7 Hz, 1H, H_β_), 7.62 (d, *J* = 8.7 Hz, 2H, H_Ar_), 7.44 (d, *J* = 15.6 Hz, 1H, H_α_), 7.30 (d, *J* = 7.8 Hz, 2H, H_Ar_), 6.96 (d, *J* = 8.9 Hz, 2H, H_Ar_), 6.38 (s, 1H, NH), 4.50 (s, 2H, CH_2_), 3.88 (m, 1H, CH), 2.43 (s, 3H, CH_3_), 1.95–1.92 (m, 2H, CH_2_), 1.74–1.69 (m, 2H, CH_2_), 1.65–1.61 (m, 1H, CH_eq_), 1.43–1.34 (m, 2H, CH_2_), 1.24–1.16 (m, 3H, CH_2_, CH_ax_) ppm; ^13^C NMR (126 MHz, CDCl_3_) δ = 190.0 (C=O_ketone_), 166.7 (C=O_amide_), 159.0, 143.7, 135.8, 130.4, 129.4, 129.2, 128.7, 120.8, 115.2 (7 × C_Ar,_ C_β,_ C_α_), 67.5 (CH_2_), 48.0 (CH), 33.1, 25.5, 24.9 (3 × CH_2_), 21.8 (CH_3_) ppm; IR (ATR) ν = 3381 (H–N), 3051 (H–C=C), 2954, 2911 (H_alkanic_), 1672 (C=O_amide_), 1650 (C=O_ketone_), 1600, 1521 (C=C), 1248, 1226, 1168, 1065, 1012 (C–O/N), 968 (H*_trans_*), 815, 750 (Ar) cm^−1^; HRMS (ESI) calculated for C_24_H_28_NO_3_ ([M + H]^+^) 378.2064, found 378.2061.

(*E*)-*N*-Cyclohexyl-2-(4-(3-(4-methoxyphenyl)-3-oxoprop-1-enyl)phenoxy)acetamide (**4b**): Yield 80%; *Rf* 0.45 (hexane:AcOEt 1:1); pale yellow solid; m.p. 135–137 °C; ^1^H NMR (500 MHz, CDCl_3_) δ = 8.03 (d, *J* = 8.9 Hz, 2H, H_Ar_), 7.76 (d, *J* = 15.6 Hz, 1H, H_β_), 7.62 (d, *J* = 8.7 Hz, 2H, H_Ar_), 7.45 (d, *J* = 15.4 Hz, 1H, H_α_), 6.99–6.95 (m, 4H, H_Ar_), 6.36 (s, 1H, NH), 4.50 (s, 2H, CH_2_), 3.91–3.85 (m, 4H, CH_3_, CH), 1.95–1.91 (m, 2H, CH_2_), 1.74–1.70 (m, 2H, CH_2_), 1.66–1.60 (m, 1H, CH_eq_), 1.42–1.35 (m, 2H, CH_2_), 1.23–1.16 (m, 3H, CH_2_, CH_ax_) ppm; ^13^C NMR (126 MHz, CDCl_3_) δ = 188.7 (C=O_ketone_), 166.7 (C=O_amide_), 163.5, 158.9, 143.2, 131.3, 130.8, 130.3, 129.3, 120.6, 115.2, 113.9 (8 × C_Ar,_ C_β,_ C_α_), 67.5 (CH_2_), 55.6 (CH_3_), 48.0 (CH), 33.1, 25.5, 24.9 (3 × CH_2_) ppm; IR (ATR) ν = 3393 (H–N), 3024 (H–C=C), 2911, 2847 (H_alkanic_), 1672 (C=O_amide_), 1645 (C=O_ketone_), 1601, 1505 (C=C), 1253, 1205, 1162, 1108, 1076 (C–O/N), 974 (H*_trans_*), 835, 812, 732 (Ar) cm^−1^; HRMS (ESI) calculated for C_24_H_28_NO_4_ ([M + H]^+^) 394.2013, found 394.2011.

(*E*)-2-(4-(3-(4-Bromophenyl)-3-oxoprop-1-enyl)phenoxy)-*N*-cyclohexylacetamide (**4c**): Yield 74%; *Rf* 0.58 (hexane:AcOEt 1:1); pale yellow solid; m.p. 180–182 °C; ^1^H NMR (500 MHz, CDCl_3_) δ = 7.87 (d, *J* = 8.5 Hz, 2H, H_Ar_), 7.77 (d, *J* = 15.7 Hz, 1H, H_β_), 7.65–7.61 (m, 4H, H_Ar_), 7.38 (d, *J* = 15.6 Hz, 1H, H_α_), 6.97 (d, *J* = 8.9 Hz, 2H, H_Ar_), 6.37 (s, 1H, NH), 4.51 (s, 2H, CH_2_), 3.91–3.84 (m, 1H, CH), 1.95–1.91 (m, 2H, CH_2_), 1.74–1.70 (m, 2H, CH_2_), 1.65–1.61 (m, 1H, CH_eq_), 1.40–1.37 (m, 2H, CH_2_), 1.23–1.16 (m, 3H, CH_2_, CH_ax_) ppm; ^13^C NMR (126 MHz, CDCl_3_) δ = 189.3 (C=O_ketone_), 166.6 (C=O_amide_), 159.2, 144.7, 137.1, 132.0, 130.5, 130.1, 128.9, 127.9, 120.1, 115.3 (8 × C_Ar,_ C_β,_ C_α_), 67.5 (CH_2_), 48.1 (CH), 33.1, 25.5, 24.9 (3 × CH_2_) ppm; IR (ATR) ν = 3385 (H–N), 3008 (H–C=C), 2917, 2831 (H_alkanic_), 1679 (C=O_amide_), 1655 (C=O_ketone_), 1600, 1521 (C=C), 1248, 1216, 1168, 1060, 1027 (C–O/N), 974 (H*_trans_*), 802, 716 (Ar) cm^−1^; HRMS (ESI) calculated for C_23_H_25_BrNO_3_ ([M + H]^+^) 442.1012, found 442.1003.

### 4.5. Reference Drug

AmB (Amphotericin B; Cristália, São Paulo, Brazil) was used as a positive control due to its well-established antileishmanial activity.

### 4.6. Leishmania Culture Conditions

Promastigote forms of *L. infantum* [IOC/L0579 (MHOM/BR/1974/PP75)] were cultured in Schneider’s medium (pH 7.0), supplemented with 20% fetal bovine serum (FBS), 1% human male urine, and 1% antibiotic solution (penicillin 200 U/mL and streptomycin 0.1 mg/mL), and maintained at 26 ± 1 °C (B.O.D SL 200—SOLAB, Piracicaba, SP, BR). The generation of extracellular axenic amastigote forms of *L. infantum* was based on protocols previously described by Debrabant et al. (2004) [40], with modifications that included the use of Schneider’s medium adjusted to pH 5.5 and incubation at 37 °C (CO_2_ Series 3517-2, Cornelius, OR, USA).

### 4.7. In Vitro Leishmanicidal Activity in Promastigote and Amastigote Forms of L. infantum

The antileishmanial activity of compounds against metacyclic promastigote populations was assessed using the MTT (ACROS, Livingston, NJ, USA) assay for cell viability analysis. During testing, *L. infantum* (IOC/L0579) cultures were maintained in vitro in supplemented Schneider’s (Sigma-Aldrich, St. Louis, MO, USA) medium (pH 7.0) at 26 ± 1 °C in a biological oxygen demand (BOD) incubator.

In 96-well plates, initial screening was performed with serial dilutions (400 to 3.12 μM) of compounds **3a**–**c** and **4a**–**c** in duplicate. Compounds **4a**–**b**, which demonstrated superior leishmanicidal activity, were subsequently tested in three independent triplicates with serial dilutions (100 to 0.78 μM). AmB was used as a positive control due to its established leishmanicidal activity, tested at concentrations ranging from 10 to 0.078 μM. Following 72 h-treatment of promastigotes and 24 h-treatment of amastigotes (accounting for the stationary phase culture characteristics), MTT was added for 4 h, followed by overnight incubation with sodium dodecyl sulfate (SDS) for spectrophotometric (Biotek model ELx800, Winooski, VT, USA) reading at 540 nm.

### 4.8. Cytotoxicity in Human Erythrocytes and Peripheral Blood Mononuclear Cells (PBMCs) Assay

The hemolytic activity of the compounds **4a**–**b** was evaluated using an adaptation of the method described by Van de Ven (2012) [41]. Blood samples were adjusted to a 5% erythrocyte concentration, taking into account the hematocrit differences between males (40–50% ≈ 45%) and females (35–45% ≈ 40%). Compounds were added to 96-well plates at serial dilutions (100 to 0.78 µM), while AmB was tested at concentrations ranging from 400 to 3.12 µM as a reference. After 1 h of incubation at 37 °C in a 5% CO_2_ incubator, 100 µL of phosphate-buffered saline (PBS) was added to each well. Plates were centrifuged (112× *g*, 10 min, 25 °C), and supernatants were transferred to a mirrored 96-well plate. Hemoglobin release, reflecting erythrocyte lysis, was quantified by measuring absorbance at 540 nm. The assay was performed in three independent experiments, with results expressed as mean hemolytic concentration (HC_50_). Ultrapure water (Direct-Q UV, Guyancourt, France) served as the positive control (100% hemolysis).

For the PBMCs assay, blood samples from three healthy volunteers were processed using Ficoll density gradient centrifugation (980× *g*) for 20 min at 25 °C. The PBMCs layer was collected, washed with sterile 1× PBS (300× *g*, 10 min, 4 °C), and resuspended in RPMI medium (Sigma-Aldrich) supplemented with 10% FBS and 1% antibiotic. Adjusted PBMCs were plated in 96-well plates and incubated with serial dilutions of the compounds (1000 to 7.8 µM) and AmB (100 to 0.78 µM) for 24 h (37 °C, 5% CO_2_). MTT was then added for 4 h, followed by centrifugation (300× *g*, 10 min) at 4 °C, supernatant removal, and DMSO addition for spectrophotometric reading at 540 nm.

### 4.9. In Silico Assays

Based on the in vitro assays, compounds **4a**–**b** underwent in silico evaluation. To assess affinity through molecular docking, three-dimensional structures of selected target enzymes were retrieved from the Protein Data Bank (PDB) [42], with inclusion criteria of crystallographic resolution < 3.0 Å and the presence of catalytically relevant cofactors. Two key metabolic targets in *Leishmania* were prioritized: the Deubiquitinase (DUB16) (PDB ID: 9I77) [43] and Tryparedoxin Peroxidase I (TXNPx) (PDB ID: 3TUE) [44]. Protein preparation was conducted in UCSF Chimera (version 1.19) [23] by removing crystallographic water molecules and non-essential ligands, while retaining cofactors essential for enzymatic activity. To ensure accurate electrostatic interaction calculations, all hydrogens were added to the protein structure, followed by the merging of non-polar hydrogens and the assignment of Kollman charges using AutoDock Tools (version 1.5.7) [24]. The chalcone derivatives were generated from SMILES strings, converted into 3D coordinates with OpenBabel (version 3.1.1) [25], and subjected to geometry optimization. Docking simulations were carried out using AutoDock Vina (version 1.2.7) [45], with the search space defined around the reported active sites of each target. Binding affinities were expressed in kcal·mol^−1^, and the best-ranked poses were examined in PyMOL (version 3.1.6.1) [26].

To further characterize binding, intermolecular interactions of the selected poses were analyzed in Discovery Studio Visualizer (Dassault Systèmes, 2002, version 3.0) [27], which enabled the classification of hydrogen bonds, hydrophobic contacts, and electrostatic interactions between ligand and active-site residues. Pharmacokinetic properties were predicted using the complementary platforms pkCSM (web-tool) [28] and SwissADME (web-tool) [29]. Toxicological endpoints were assessed using ProTox-II (web tool) [30], which included acute oral toxicity (LD_50_, mg/kg) and additional endpoints.

### 4.10. Synergistic Activity

The selected compound (**4b**) was tested in combination with AmB at a fixed 1:5 ratio against axenic amastigotes (0.156/0.78; 0.312/1.56; 0.62/3.1; 1.25/6.25; and 2.5/12.5 µM) and PBMCs (6.25/31.25; 12.5/62.5; 25/125; 50/250; and 100/500 µM), following previously described cell viability protocols. The nature of the drug interaction (synergistic, additive, or antagonistic) was evaluated using the Chou–Talalay method implemented in CompuSyn software (ComboSyn Incorporated, version 1.0.1, 2006–2023). This approach calculates the Combination Index (CI) based on dose–response data fitted to the median-effect equation, with parameters including Dm (median-effect dose), m (slope of the dose–effect curve), and r (linear correlation coefficient). To characterize the interaction at different effect levels, CI values were determined for the following effective doses: ED_50_, ED_75_, and ED_90_, corresponding to 50%, 75%, and 90% inhibition of cell viability, respectively. Interactions were interpreted as synergistic when CI < 1, additive when CI = 1, and antagonistic when CI > 1. Additionally, the dose reduction index (DRI) was calculated to estimate the amount of dose reduction that could be achieved for each drug in combination while maintaining the same level of efficacy; DRI values greater than 1 indicate a favorable dose reduction [46].

### 4.11. Statistical Analysis

Values of 50% inhibitory concentration (IC_50_), 50% cytotoxic concentration (CC_50_), and 50% hemolytic concentration (HC_50_) were calculated using the GraphPad Prism software (version 6.0; San Diego, CA, USA). Statistical analysis was performed using nonlinear regression (curve fit), and significant differences (*p* ≤ 0.05 *) were determined by one-way ANOVA and Tukey’s post hoc test, and a 95% Confidence Interval (CI) was used to assess variance across cell populations.

## 5. Conclusions

In conclusion, a novel series of chalcone–acetamides was synthesized and evaluated for their antileishmanial potential. Compounds bearing a cyclohexyl group (**4a**–**c**) demonstrated superior biological activity compared to their phenyl-substituted analogs (**3a**–**c**), with compound **4b** exhibiting the most promising results in terms of anti-*L. infantum* activity and selectivity. In silico studies provided a plausible mechanistic basis for its efficacy, predicting affinity with two critical parasite survival pathways associated with enzymes: ubiquitination homeostasis (via DUB16) and antioxidant defense (via TXNPx). While the inactivity of compounds **3a**–**c** remains inconclusive, it highlights the need to further investigate the role of physicochemical properties in parasite interaction. Additionally, compound **4b** displayed a synergistic interaction with AmB at higher inhibitory concentrations, potentially allowing for a dose reduction of the reference drug without increasing cytotoxicity. These findings support compound **4b** as a potential lead compound for further preclinical development, either as monotherapy or in combination strategies. Overall, this work reinforces the therapeutic relevance of chalcone scaffolds and encourages continued exploration of their chemical space for the treatment of neglected tropical diseases.

## Figures and Tables

**Table 1:** CI = Confidence Interval. SI = Selectivity Index. CC_50_ = 50% Cytotoxic Concentration. IC_50_ = 50% Inhibitory Concentration.

Compound	CC_50_ (CI)	Promastigotes IC_50_ (CI)	SI	Amastigotes IC_50_ (CI)	SI
**4a**	157.1 μM (105.6 to 241.8)	12.9 µM (9.81 to 17.01)	12.2	19.65 µM (11.9 to 33.2)	8
**4b**	505.4 μM (333.6 to 914.3)	7.02 µM (3.73 to 15.48)	72	3.4 µM (2.99 to 3.9)	148.6
AmB	29.9 µM (19.15 to 53.4)	0.87 µM (0.56 to 1.41)	34.4	0.89 µM (0.43 to 2.1)	33.6

**Table 2:** In silico pharmacokinetic and toxicological properties of compounds **4a**–**b**. Parameters include predicted acute oral toxicity in rats (LD_50_), Caco-2 cell permeability, inhibition of P-glycoprotein (P-gp), inhibition of Cytochrome P450 2D6 (CYP2D6), calculated octanol–water partition coefficient (Log P_o/w_), Topological Polar Surface Area (TPSA), and the number of violations of Lipinski’s rule of five.

Compound	Class	LD_50_ (mg/kg^−1^)	Caco-2	P-gp Inhibitor	CYP2D6 Inhibitor	Log P_o/w_	TPSA	Lipinski Violations
**4a**	4	1600	1.474	Yes	No	4.35	55.4	0
**4b**	4	1600	0.651	Yes	No	4.02	64.63	0

## Data Availability

The original contributions presented in this study are included in the article/Supplementary Materials. Further inquiries can be directed to the corresponding author.

## Supplementary Materials

The following supporting information can be downloaded at: https://www.mdpi.com/article/10.3390/antibiotics14111123/s1, Figure S1. ^1^H NMR spectrum (500 MHz, CDCl_3_) of compound **3a**. Figure S2. ^13^C NMR spectrum (126 MHz, CDCl_3_) of compound **3a**. Figure S3. IR spectrum (ATR) of compound **3a**. Figure S4. HRMS spectrum (ESI) of compound **3a**. Figure S5. ^1^H NMR spectrum (400 MHz, CDCl_3_) of compound **3b**. Figure S6. ^13^C NMR spectrum (101 MHz, CDCl_3_) of compound **3b**. Figure S7. IR spectrum (KBr) of compound **3b**. Figure S8. HRMS spectrum (ESI) of compound **3b**. Figure S9. ^1^H NMR spectrum (500 MHz, CDCl_3_) of compound **3c**. Figure S10. ^13^C NMR spectrum (126 MHz, CDCl_3_) of compound **3c**. Figure S11. IR spectrum (ATR) of compound **3c**. Figure S12. HRMS spectrum (ESI) of compound **3c**. Figure S13. ^1^H NMR spectrum (500 MHz, CDCl_3_) of compound **4a**. Figure S14. ^13^C NMR spectrum (126 MHz, CDCl_3_) of compound **4a**. Figure S15. IR spectrum (ATR) of compound **4a**. Figure S16. HRMS spectrum (ESI) of compound **4a**. Figure S17. ^1^H NMR spectrum (500 MHz, CDCl_3_) of compound **4b**. Figure S18. ^13^C NMR spectrum (126 MHz, CDCl_3_) of compound **4b**. Figure S19. IR spectrum (ATR) of compound **4b**. Figure S20. HRMS spectrum (ESI) of compound **4b**. Figure S21. ^1^H NMR spectrum (500 MHz, CDCl_3_) of compound **4c**. Figure S22. ^13^C NMR spectrum (126 MHz, CDCl_3_) of compound **4c**. Figure S23. IR spectrum (ATR) of compound **4c**. Figure S24. HRMS spectrum (ESI) of compound **4c**.
