# Supplementary material for: Novel Chalcone Derivatives as Anti-Leishmania infantum Agents with Potential Synergistic Activity and In Silico Insights
Source: Antibiotics (Basel). 2025 Nov 7;14(11):1123. doi: 10.3390/antibiotics14111123 (PMC12649483; doi:10.3390/antibiotics14111123)
Supplement: Supplementary file 1 [file antibiotics-14-01123-s001.zip › antibiotics-3936503-supplementary.docx]

Novel Chalcone Derivatives as Anti-*Leishmania infantum* Agents with Potential Synergistic Activity and In
Silico Insights

Ana Letícia Monteiro Fernandes ^1^, Abraão Pinheiro Sousa ^2^, Delva Thyares Fonseca Lamec ^1^,
Leonardo Lima Cardoso ^1^, Rosália Santos Ferreira ^1^, Shayenne Eduarda Ramos Vanderley ^1^,
Petrônio Filgueiras Athayde-Filho ^2^, Gabriela Fehn Fiss ^2^ and Tatjana Souza Lima Keesen ^1,^*

^1^ Immunology Laboratory of Infectious Diseases (LABIDIC), Department of Cellular and Molecular Biology, Federal University of Paraiba, João Pessoa 58051-900, Brazil; amf.leticia@gmail.com (A.L.M.F.)

^2^ Bioenergy and Organic Synthesis Research Laboratory (LPBS), Department of Chemistry, Federal University of Paraíba, João Pessoa 58051-900, Brazil

***** Correspondence: tat.keesen@cbiotec.ufpb.br; Tel.:+55-83-32167173

**Supplementary data**

Full NMR, IR and HRMS spectra for chalcone**–**acetamides (**3a–c**, **4a–c**) are available in **Figures S1–S24**.


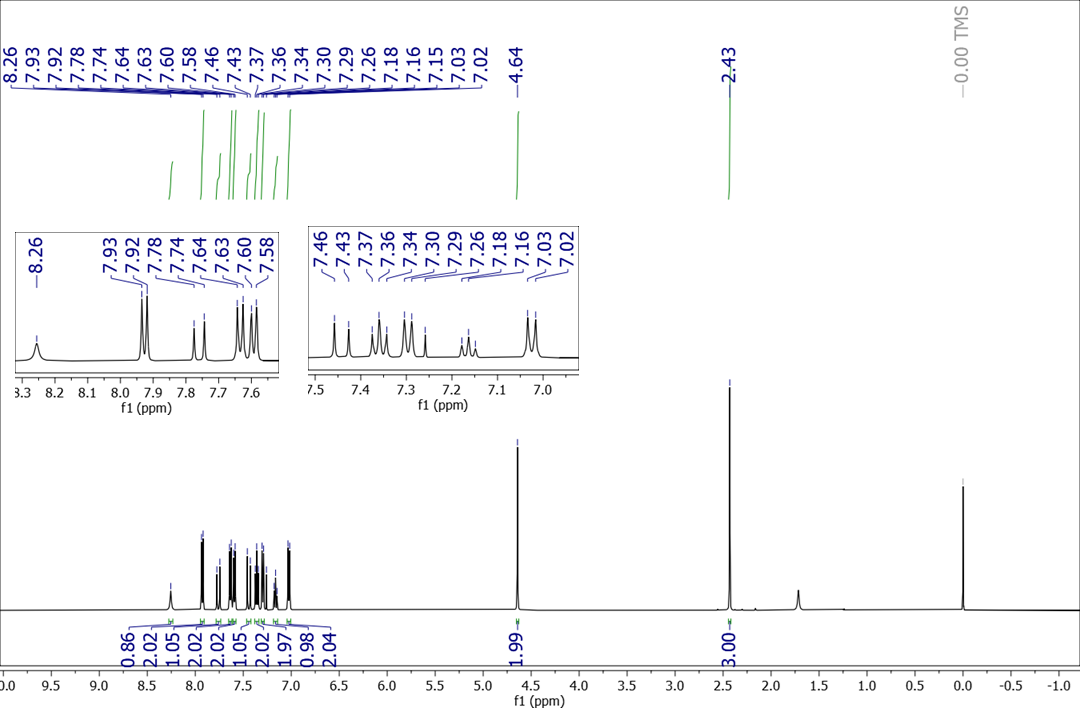


**Figure S1.** ^1^H NMR spectrum (500 MHz, CDCl_3_) of compound **3a**.


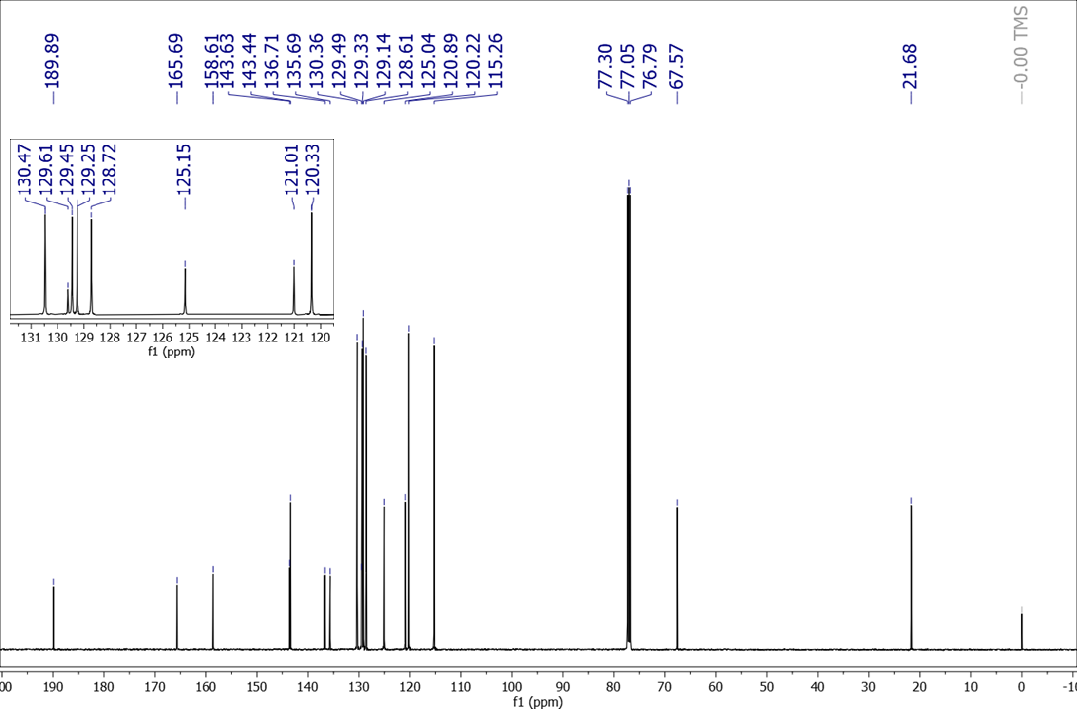


**Figure S2.** ^13^C NMR spectrum (126 MHz, CDCl_3_) of compound **3a**.


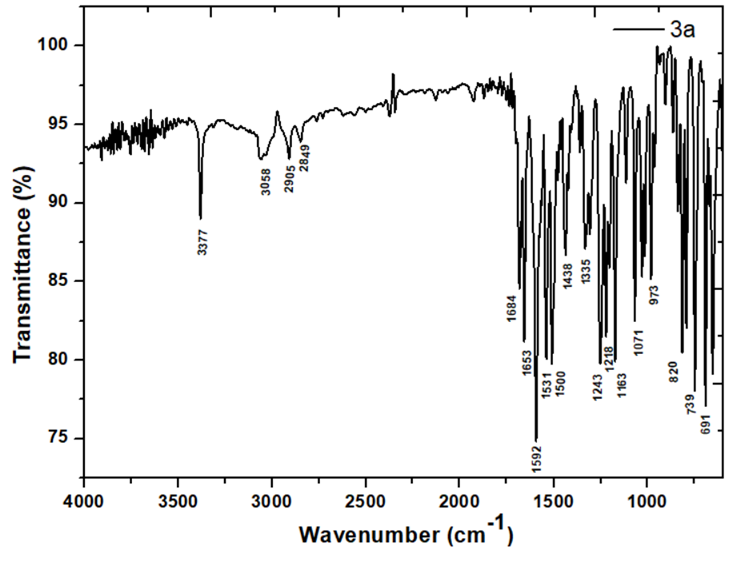


**Figure S3.** IR spectrum (ATR) of compound **3a**.


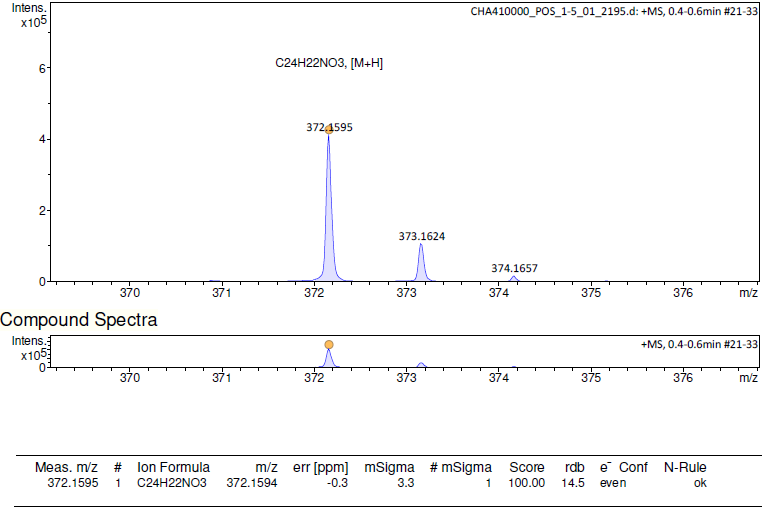


**Figure S4.** HRMS spectrum (ESI) of compound **3a**.


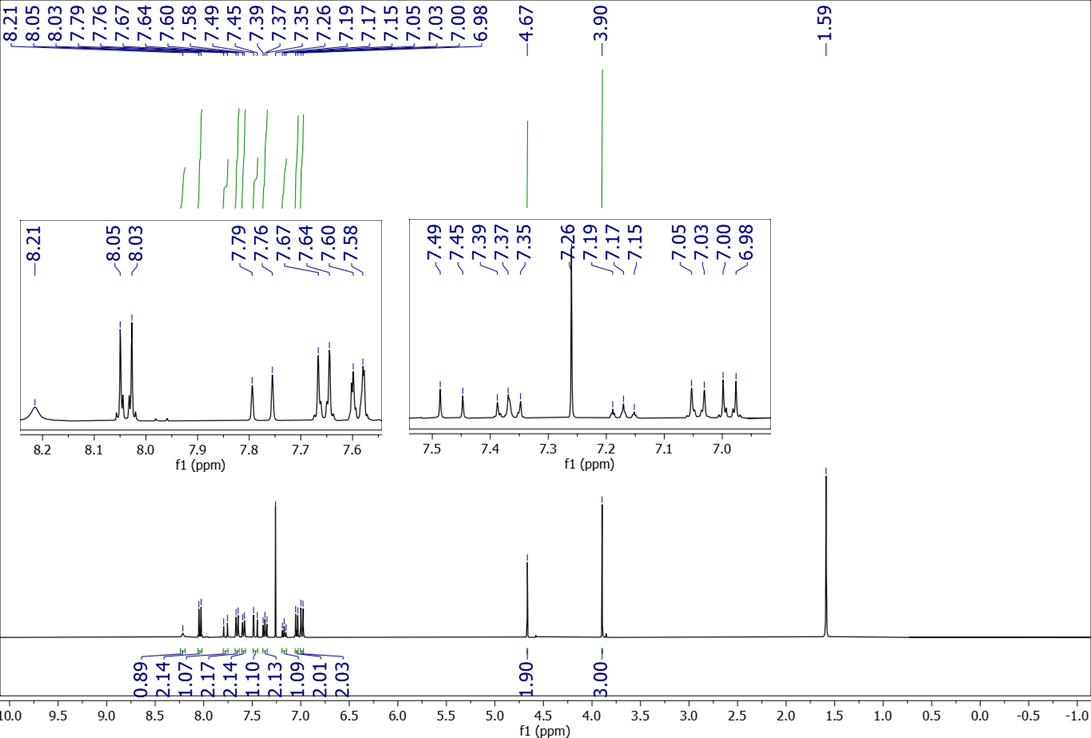


**Figure S5.** ^1^H NMR spectrum (400 MHz, CDCl_3_) of compound **3b**.


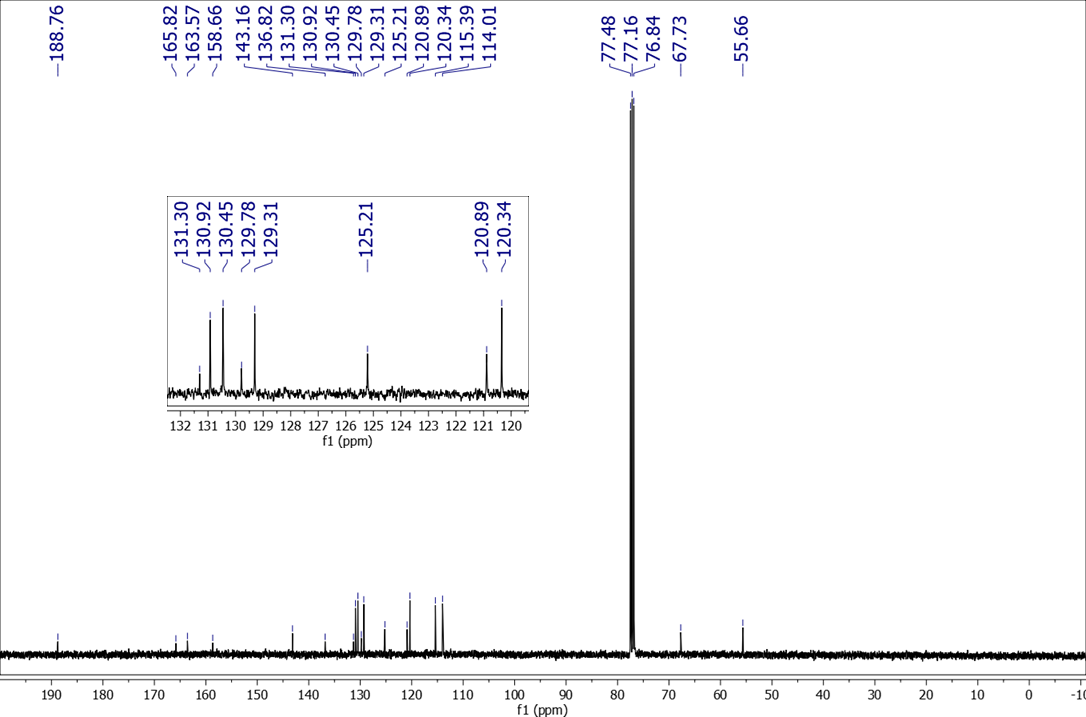


**Figure S6.** ^13^C NMR spectrum (101 MHz, CDCl_3_) of compound **3b**.


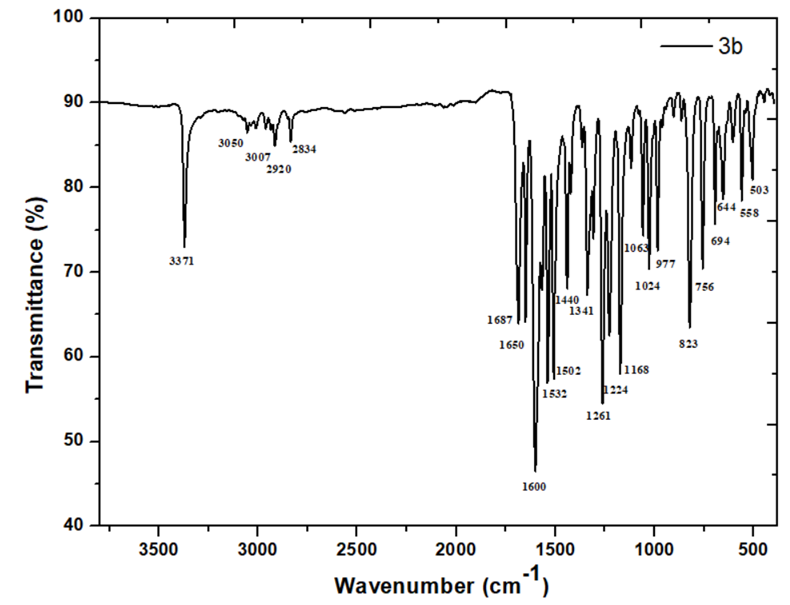


**Figure S7.** IR spectrum (KBr) of compound **3b**.


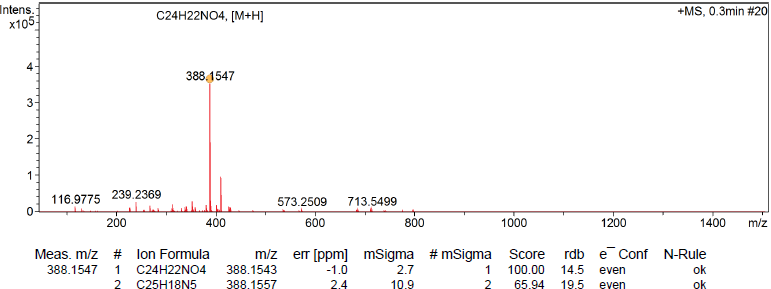


**Figure S8.** HRMS spectrum (ESI) of compound **3b**.


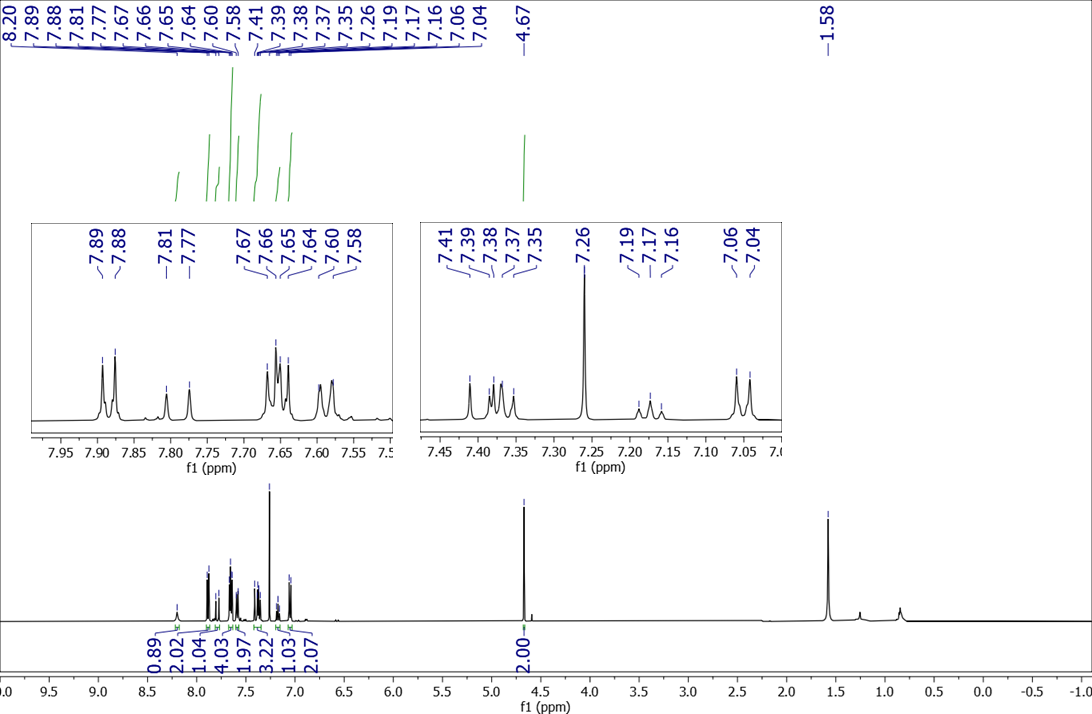


**Figure S9.** ^1^H NMR spectrum (500 MHz, CDCl_3_) of compound **3c**.


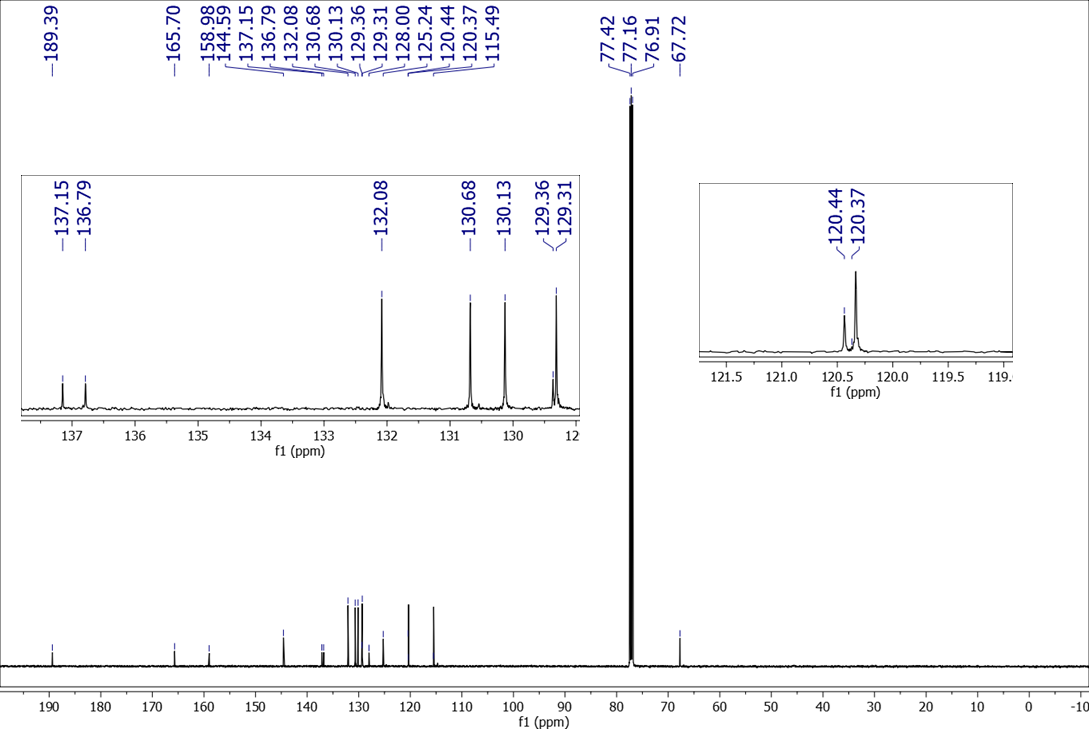


**Figure S10.** ^13^C NMR spectrum (126 MHz, CDCl_3_) of compound **3c**.


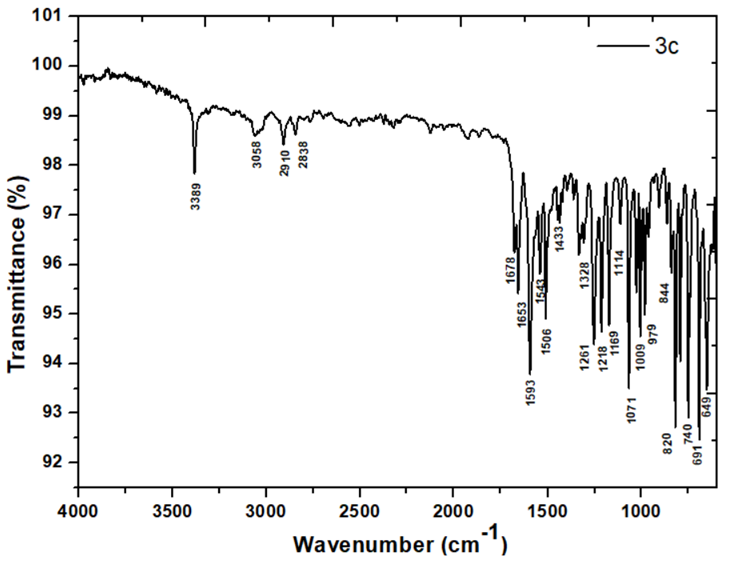


**Figure S11.** IR spectrum (ATR) of compound **3c**.


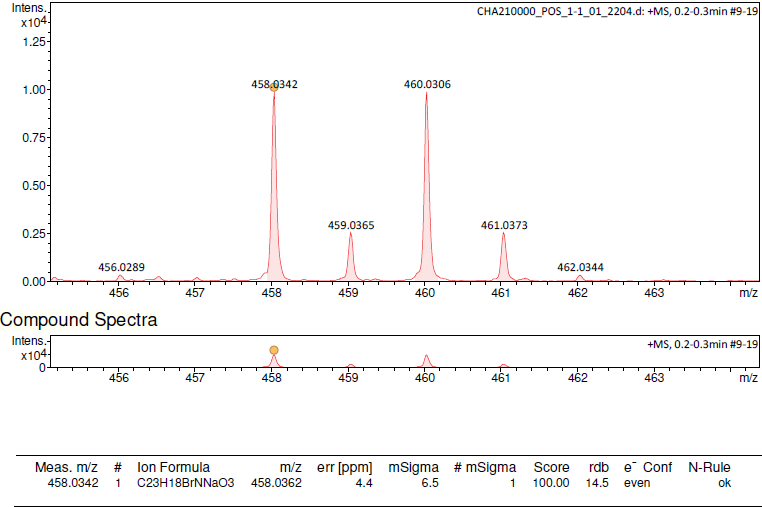


**Figure S12.** HRMS spectrum (ESI) of compound **3c**.

**
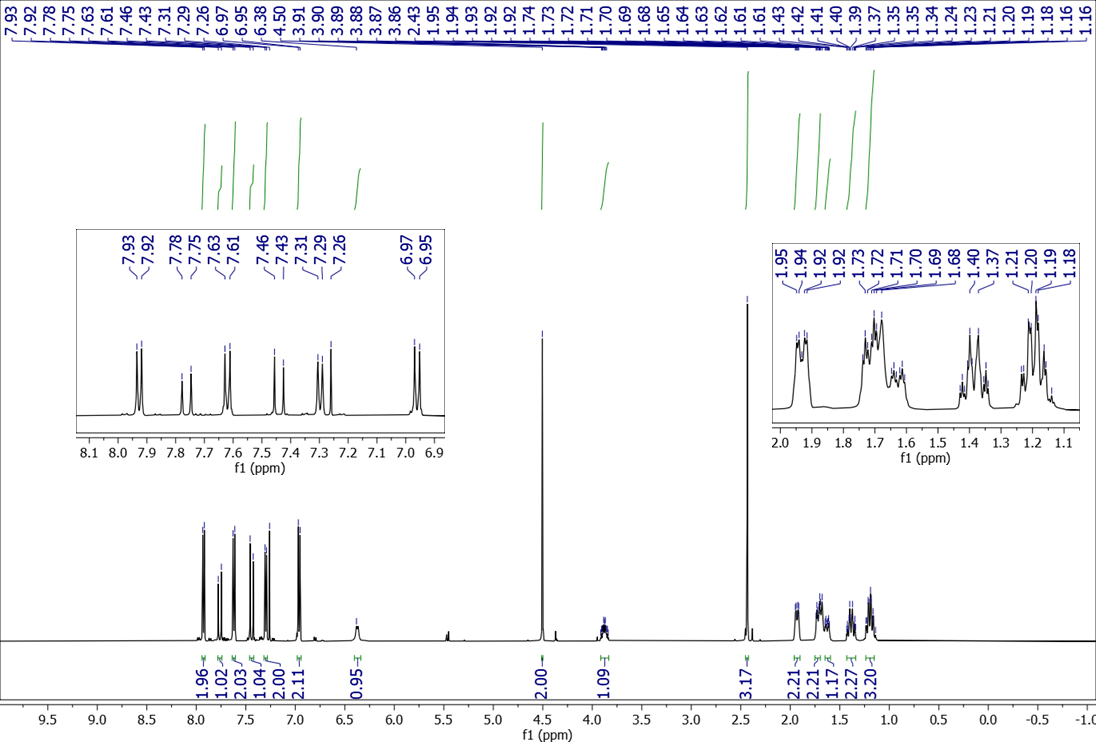
**

**Figure S13.** ^1^H NMR spectrum (500 MHz, CDCl_3_) of compound **4a**.


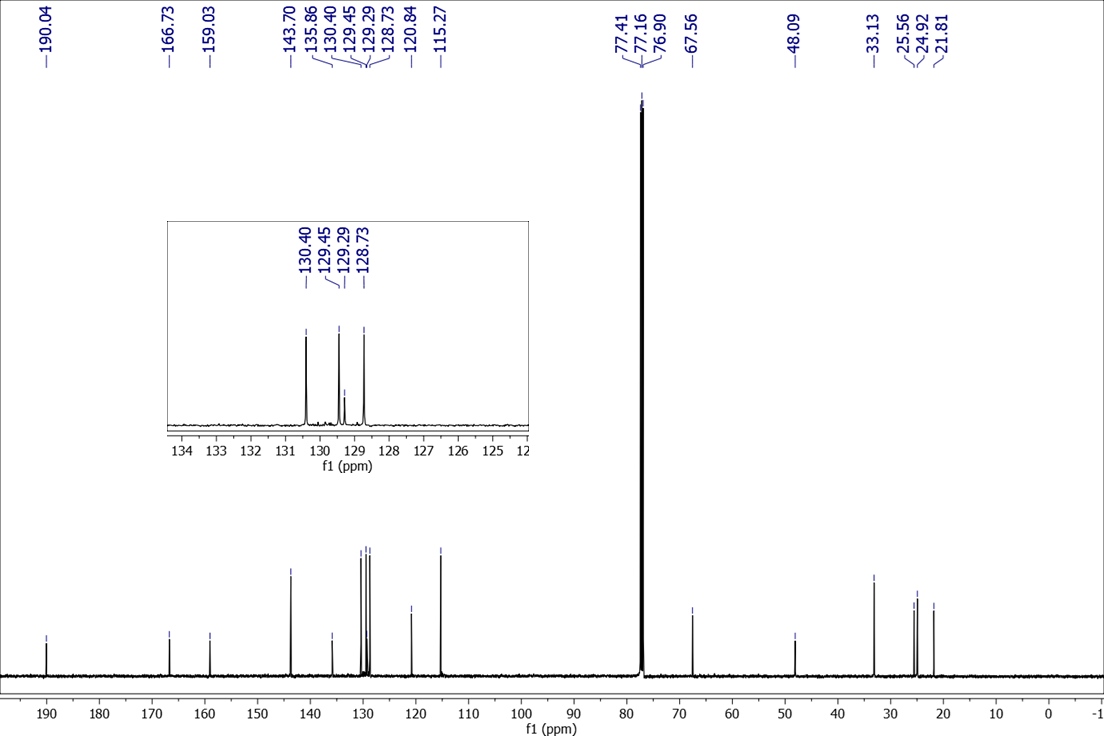


**Figure S14.** ^13^C NMR spectrum (126 MHz, CDCl_3_) of compound **4a**.


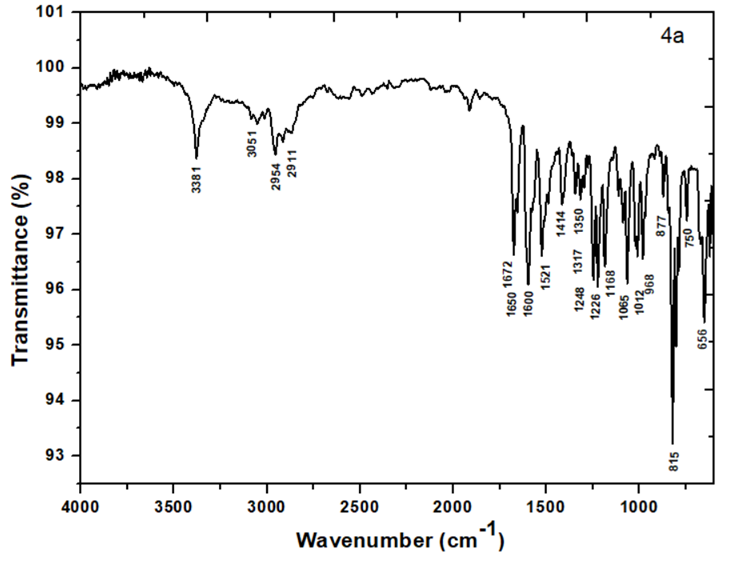


**Figure S15.** IR spectrum (ATR) of compound **4a**.


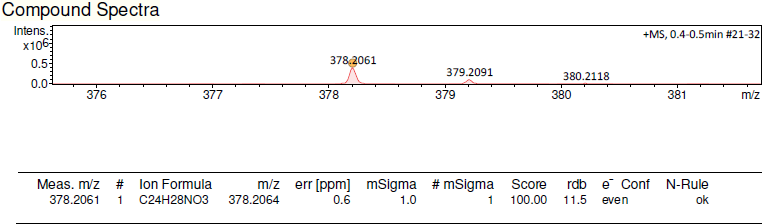


**Figure S16.** HRMS spectrum (ESI) of compound **4a**.


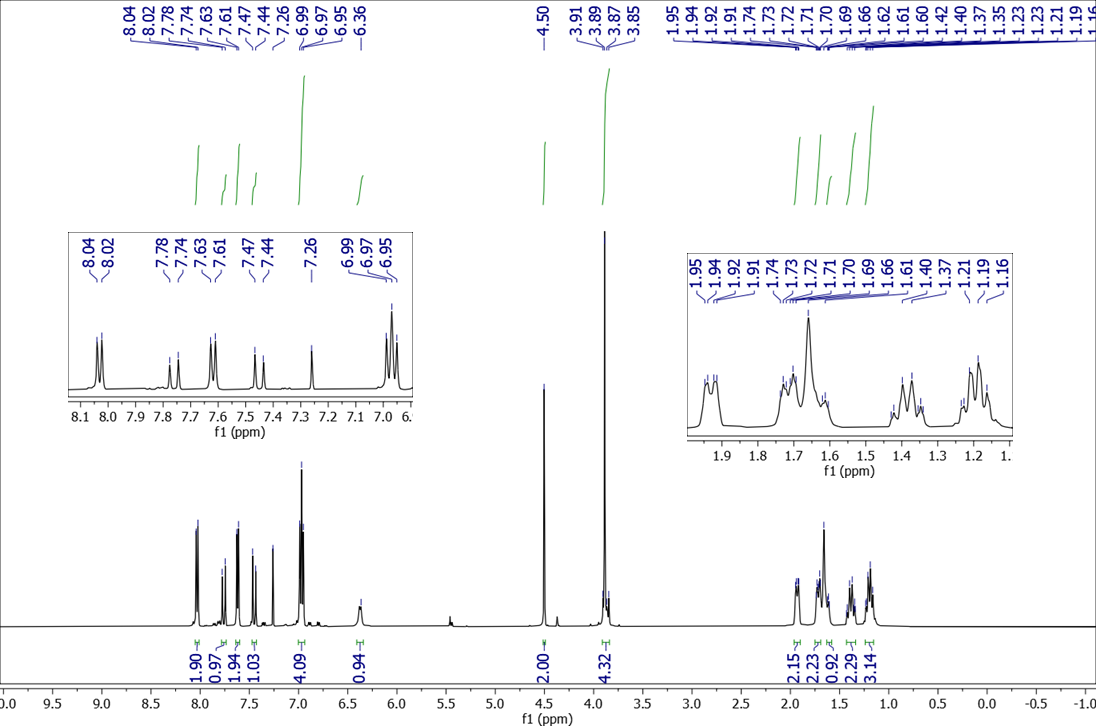


**Figure S17.** ^1^H NMR spectrum (500 MHz, CDCl_3_) of compound **4b**.


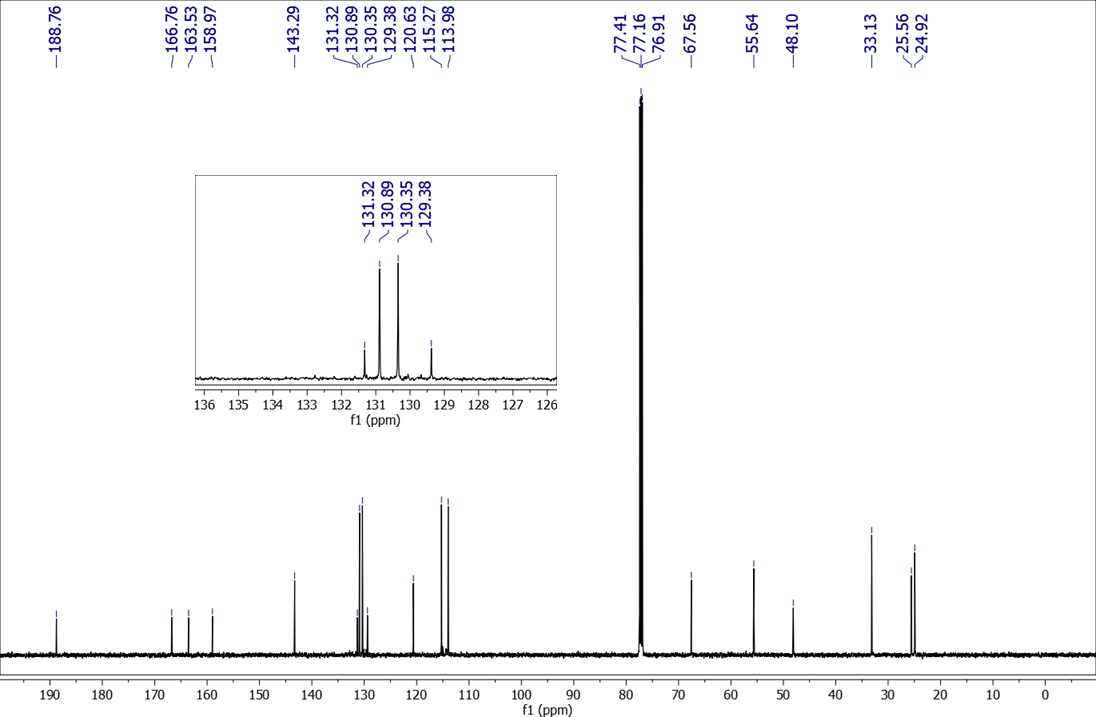


**Figure S18.** ^13^C NMR spectrum (126 MHz, CDCl_3_) of compound **4b**.


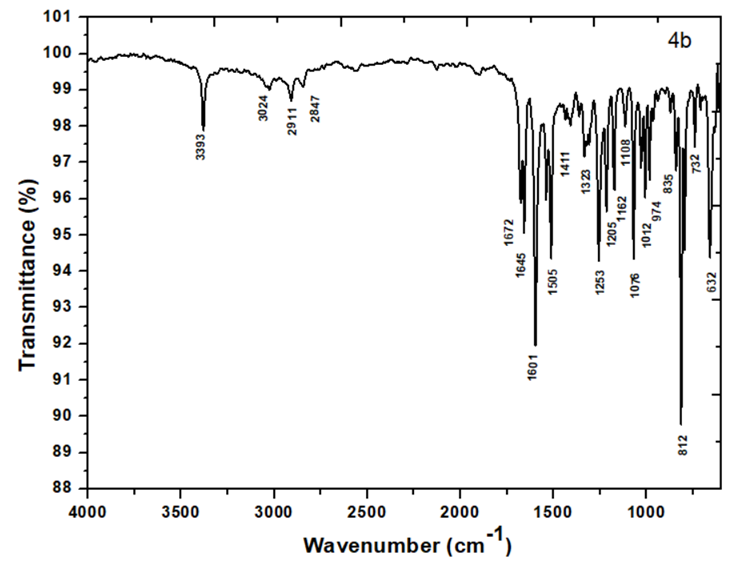


**Figure S19.** IR spectrum (ATR) of compound **4b**.


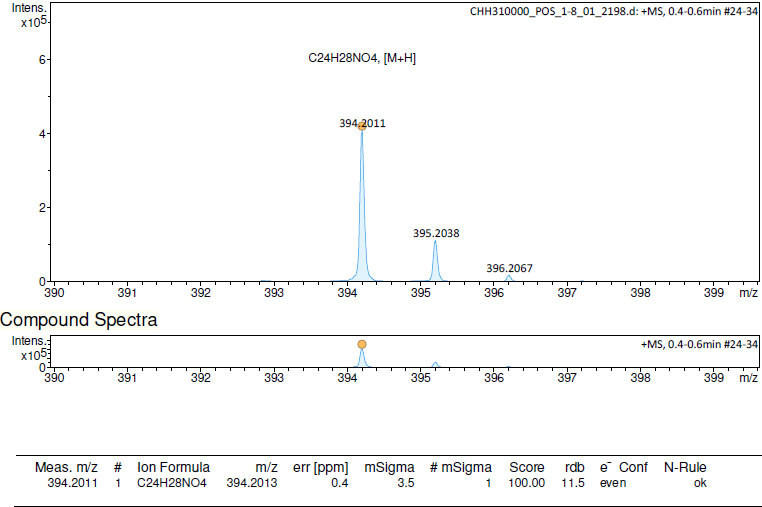


**Figure S20.** HRMS spectrum (ESI) of compound **4b**.


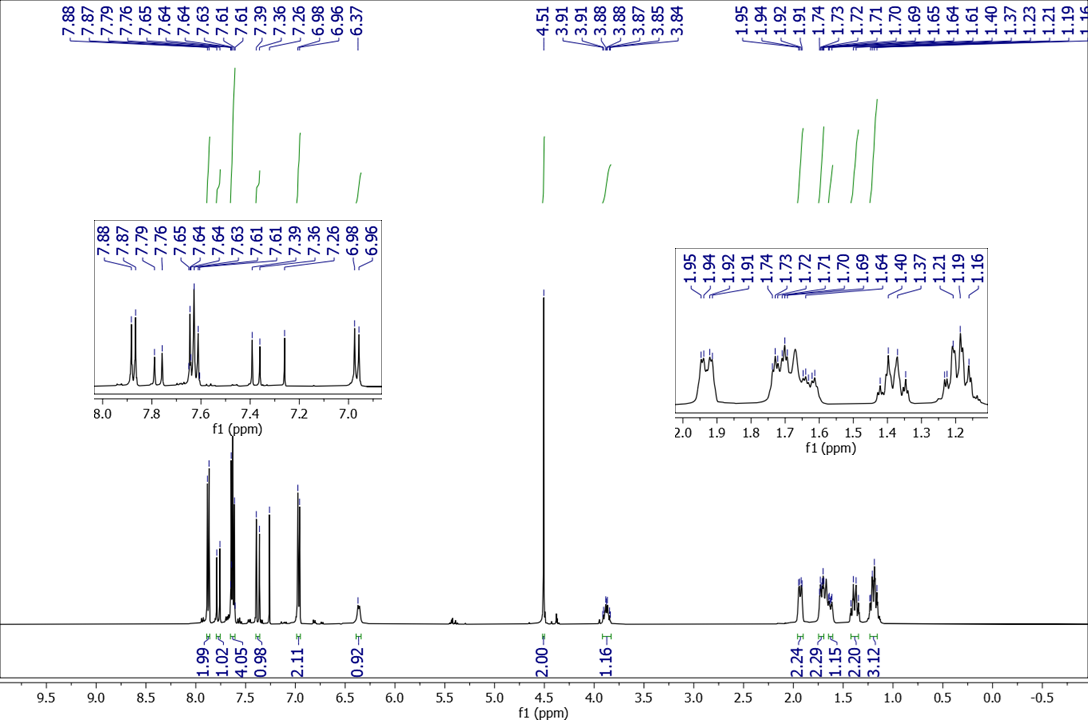


**Figure S21.** ^1^H NMR spectrum (500 MHz, CDCl_3_) of compound **4c**.


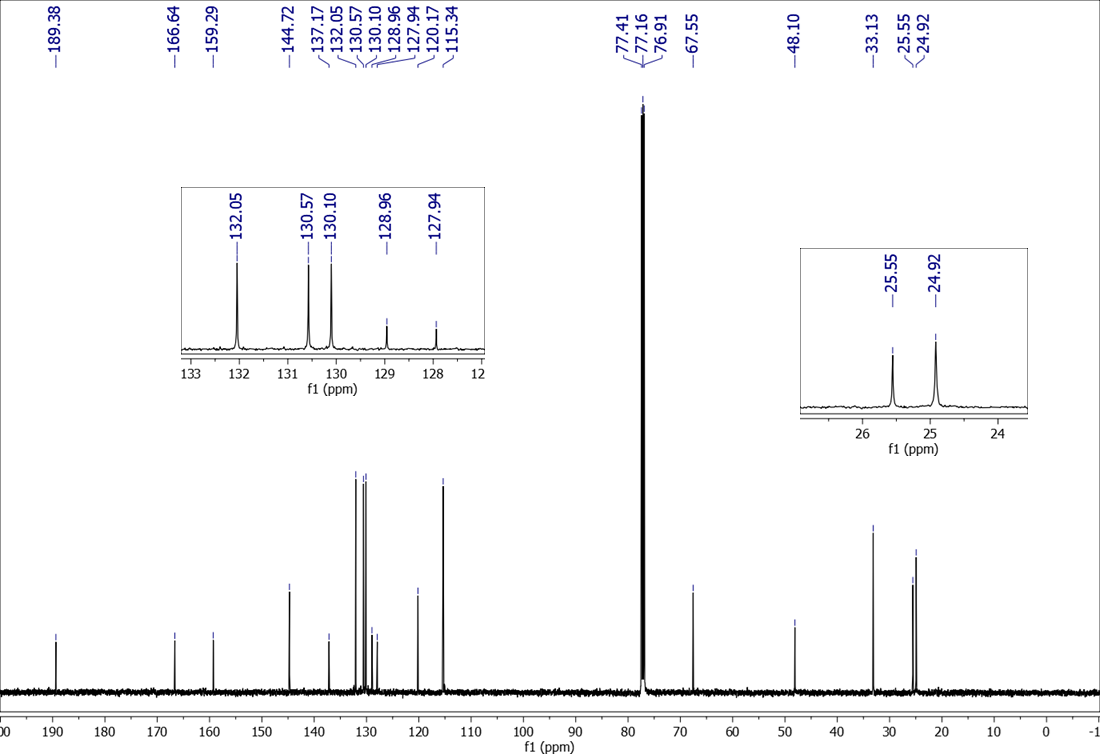


**Figure S22.** ^13^C NMR spectrum (126 MHz, CDCl_3_) of compound **4c**.


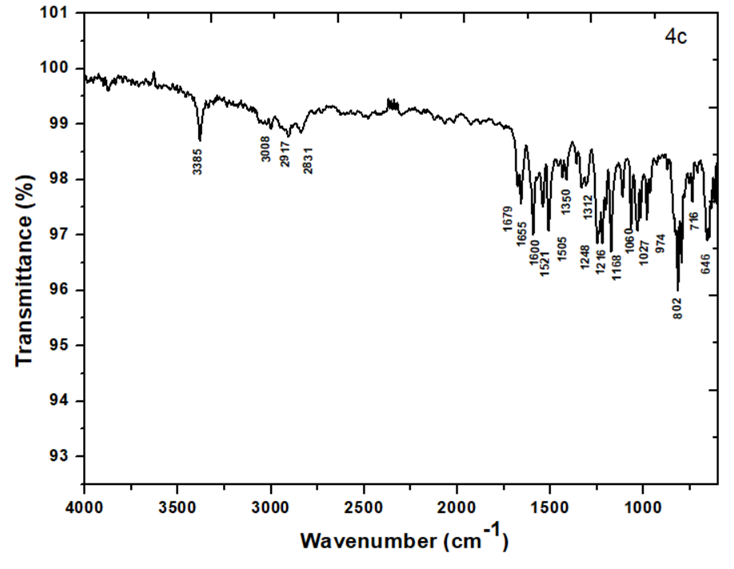


**Figure S23.** IR spectrum (ATR) of compound **4c**.


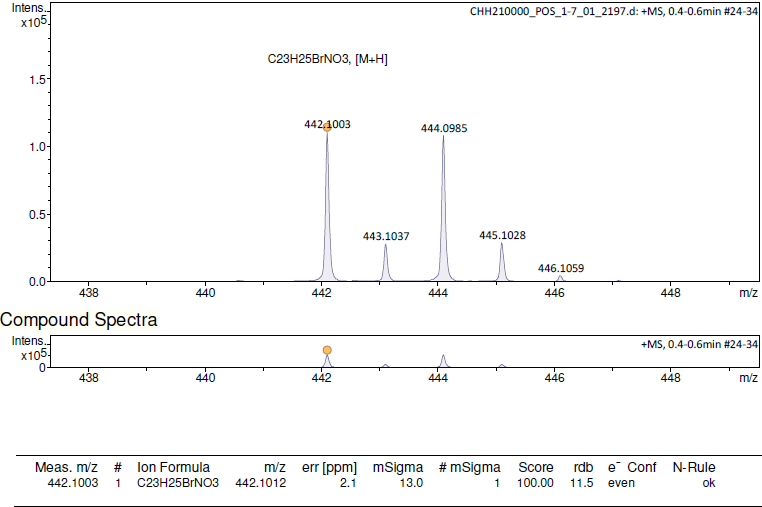


**Figure S24.** HRMS spectrum (ESI) of compound **4c**.
